# Small Deletion Variants Have Stable Breakpoints Commonly Associated with *Alu* Elements

**DOI:** 10.1371/journal.pone.0003104

**Published:** 2008-08-29

**Authors:** Adam J. de Smith, Robin G. Walters, Lachlan J. M. Coin, Israel Steinfeld, Zohar Yakhini, Rob Sladek, Philippe Froguel, Alexandra I. F. Blakemore

**Affiliations:** 1 Section of Genomic Medicine, Imperial College London, Hammersmith Hospital, London, United Kingdom; 2 Department of Epidemiology & Public Health, Imperial College London, London, United Kingdom; 3 Agilent Laboratories, Petah Tiqva, Israel; 4 Department of Human Genetics, McGill University and Génome Québec Innovation Centre, Montreal, Quebec, Canada; 5 Department of Medicine, McGill University and Génome Québec Innovation Centre, Montreal, Quebec, Canada; 6 CNRS 8090-Institute of Biology, Pasteur Institute, Lille, France; National Cancer Institute, United States of America

## Abstract

Copy number variants (CNVs) contribute significantly to human genomic variation, with over 5000 loci reported, covering more than 18% of the euchromatic human genome. Little is known, however, about the origin and stability of variants of different size and complexity. We investigated the breakpoints of 20 small, common deletions, representing a subset of those originally identified by array CGH, using Agilent microarrays, in 50 healthy French Caucasian subjects. By sequencing PCR products amplified using primers designed to span the deleted regions, we determined the exact size and genomic position of the deletions in all affected samples. For each deletion studied, all individuals carrying the deletion share identical upstream and downstream breakpoints at the sequence level, suggesting that the deletion event occurred just once and later became common in the population. This is supported by linkage disequilibrium (LD) analysis, which has revealed that most of the deletions studied are in moderate to strong LD with surrounding SNPs, and have conserved long-range haplotypes. Analysis of the sequences flanking the deletion breakpoints revealed an enrichment of microhomology at the breakpoint junctions. More significantly, we found an enrichment of *Alu* repeat elements, the overwhelming majority of which intersected deletion breakpoints at their poly-A tails. We found no enrichment of LINE elements or segmental duplications, in contrast to other reports. Sequence analysis revealed enrichment of a conserved motif in the sequences surrounding the deletion breakpoints, although whether this motif has any mechanistic role in the formation of some deletions has yet to be determined. Considered together with existing information on more complex inherited variant regions, and reports of *de novo* variants associated with autism, these data support the presence of different subgroups of CNV in the genome which may have originated through different mechanisms.

## Introduction

Copy number variation represents a significant proportion of the genetic difference between apparently healthy individuals [1]–[5], with over 5000 variant loci, covering more than 18% of the euchromatic genome, currently documented [6]. Copy number variants (CNVs) have been estimated to account for at least 17.7% of heritable variation in gene expression [7], and have been associated with a number of diseases, such as autism [8], glomerulonephritis [9], and resistance to HIV [10].

CNVs vary greatly in size, with variants ranging from insertions or deletions of under 1 kb (commonly described as indels) to several Mb in length. They also vary in complexity, ranging from simple CNVs flanked by common boundaries to more complex overlapping patterns of deletion or duplication that may be observed in particular genomic regions [4]. In addition to different types of CNVs varying in complexity and size, they may also differ in their mechanism of origin. In a number of studies, associations have been reported between genomic regions enriched with CNVs and segmental duplications [4], [5], [11], which have been suggested to mediate the formation of variants by non-allelic homologous recombination (NAHR). Not all CNVs, however, are associated with these repeats: approximately half of all reported CNV sequences do not overlap segmental duplications [12]. Two recent studies suggest that the majority of CNVs are formed by another mechanism, known as non-homologous end joining (NHEJ), which is associated with microhomology rather than with long stretches of sequence identity at CNV breakpoints [13], [14]. A further difference between CNV subtypes has been observed in the extent of linkage disequilibrium (LD) between a CNV and the surrounding single nucleotide polymorphisms (SNPs); stronger LD was found between SNPs and common deletions [15], [16] than with CNVs in duplication-rich regions [17].

We have previously reported a high-resolution array CGH (aCGH) screen, for CNVs in 50 apparently healthy, French Caucasian adult males [18]. In this study, it was observed that some regions of the genome showed complex overlapping patterns of deletion or duplication, but of CNVs found in more than one individual, the majority (83%) had very consistent boundaries as determined by aCGH in unrelated individuals. The aim of the present study was to investigate the mechanism of formation of a subset of these CNVs. Sequencing across the breakpoints of 20 small, common deletions with such consistent boundaries, interrogation of these regions for the presence of repeat elements and for sequence similarity, and analysis of LD relationships with nearby SNPs, have together provided evidence concerning the origins of these CNVs and their maintenance in the general population.

## Results

### Deletion breakpoint analysis

**Figure 1:**
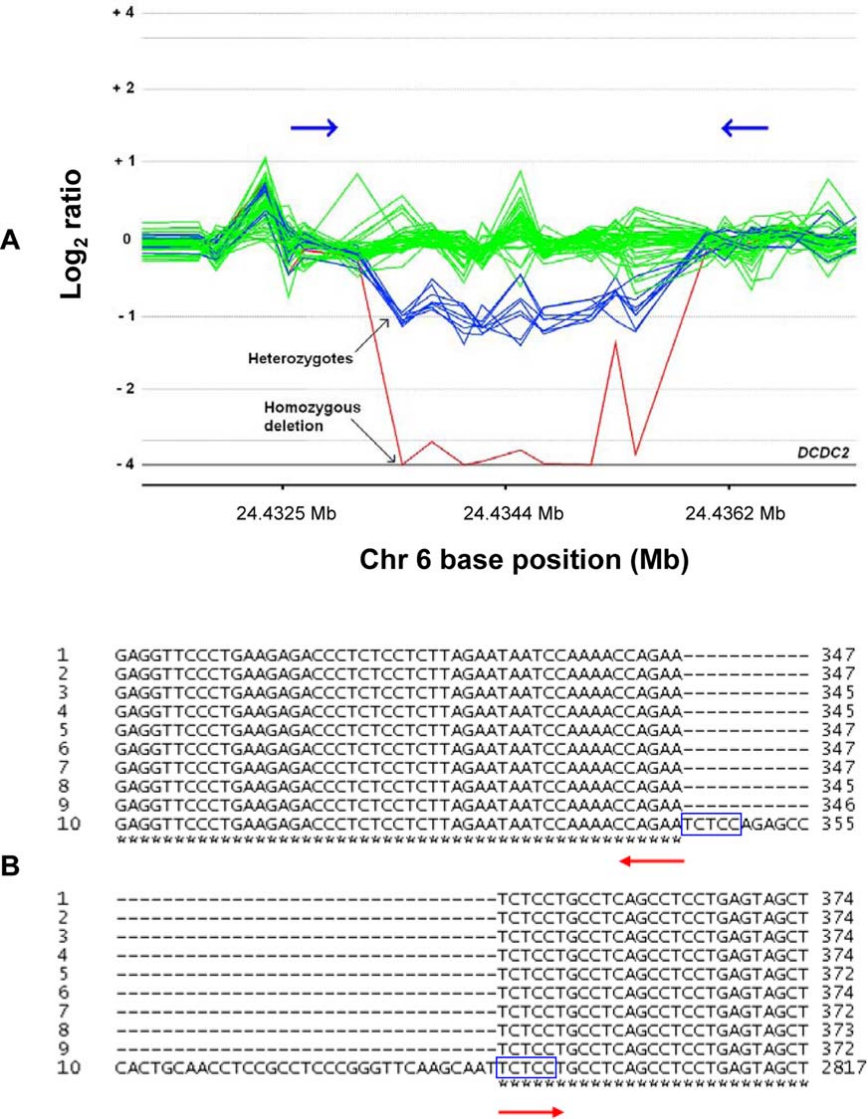
Example of deletion within *DCDC2* gene having identical sequence breakpoints in 9 samples (representing 10 chromosomes). A: CGH Analytics view of deletion detected by 9 consecutive probes within intronic region of gene *DCDC2* at chr6: 24,433,346–24,435,791 (UCSC March 2006). The superimposed log_2_ ratios for the 50 samples are plotted as a function of chromosomal position and with different colours for putatively different copy number states (undeleted samples = green, putative heterozygous deletion samples = blue, and the putative homozygous deletion sample = red). Log_2_ ratios for 8 samples are around −1 (putative heterozygous deletion compared to the reference sample) and log_2_ ratio for one sample is around −4 (putative homozygous deletion). Blue arrows indicate approximate position of PCR primers. B: Multiple sequence alignment (using ClustalW) of 9 deleted samples (rows 1–9) with reference genome sequence (row 10). Asterisks indicate where all 10 sequences are perfectly aligned around the deleted region; upstream and downstream deletion breakpoints are indicated by the red arrows. The deleted region begins 345 bp into the reference sequence, and ends at 2790 bp, thus the deletion size is 2446 bp in all 9 samples. Blue boxes indicate 5 bp sequences of microhomology between the upstream and downstream breakpoints.

Sequences immediately upstream and downstream of each deleted region were amplified by PCR, using primer pairs designed to flank the position of the deletions, as predicted by the genomic locations of the aCGH probes (see Materials and Methods). Multiple alignments of each deleted sample sequence with the human reference sequence (UCSC March 2006) [19] enabled determination of the precise size and genomic location of each deletion (see Table 1). For each of the deletions investigated, all samples shared identical sequence breakpoints at the upstream and downstream ends of the deletion, confirming the common boundaries of the aberrations as called in our aCGH data, as illustrated (Figure 1).

**Table 1:** Summary of the 20 sequenced deletion variants.

Genomic Position (deleted sequence)	Deletion size (bp)	Number of samples with deletion (≥1 copy)	Number of chromosomes sequenced	Deletion within gene	Intronic/exonic	Repeat sequences at upstream breakpoint	Repeat sequences at downstream breakpoint	Maximum R^2^ values within 100 kb window	P-values
Chr 1: 145,312,298–145,314,875	2578	3	2			Low-Complexity Repeat (AT-rich)	*Alu* element* (*Alu*Y)	0.07	0.155
Chr 2: 229,467,533–229,468,151	619	10	10			*Alu* element (*Alu*Y)		0.67	1.0E-4
Chr 3: 181,137,036–181,137,500	465	9	10	*PEX5L*	Intron 2–3			0.43	1.0E-4
Chr 4: 98,573,315–98,578,237	4923	11	11			LINE1 element	ERV1 LTR (endogenous retrovirus 1, long terminal repeat)	0.10	0.184
Chr 5: 65,479,440–65,479,975	536	2	2					0.99	1.0E-4
Chr 5: 78,145,556–78,147,626	2071	8	6	*ARSB*	Intron 6–7			0.24	0.032
Chr 6: 24,433,346–24,435,791	2446	9	10	*DCDC2*	Intron 2–3		*Alu* element (*Alu*S)	0.75	1.0E-4
Chr 6: 34,425,089–34,427,582	2494	4	3	*NUDT3*	Intron 1–2	LINE2 element		0.47	1.0E-4
Chr 6: 162,645,085–162,645,903	819+4 bp insertion	5	6	*PARK2*	Intron 1–2	LINE1 element		0.41	6.0E-3
Chr 7: 82,856,584–82,857,509	926	12	14	*SEMA3E*	Intron 14–15			0.91	1.0E-4
Chr 12: 20,859,912–20,859,936	25+3 bp insertion	26	32	*SLCO1B3*	ENSE00000822206			0.52	1.0E-4
Chr 14: 72,402,707–72,403,561	855	5	6	*DPF3*	Intron 1–2		*Alu* element (FLAM_C)	0.27	0.01
Chr 14: 72,615,524–72,616,685	1162	10	10	*RBM25*	Intron 4–5		*Alu* element* (*Alu*Y)	0.88	1.0E-4
Chr 15: 83,858,016–83,860,206	2191	14	19	*AKAP13*	Intron 3–4	LINE1 element		0.90	1.0E-4
Chr 16: 22,955,277–22,957,032	1756+6 bp insertion	44	36					0.82	1.0E-4
Chr 16: 56,282,301–56,285,908	3608	18	3			*Alu* element* (*Alu*J)	*Alu* element* (FLAM_C)	0.92	1.0E-4
Chr 16: 76,115,174–76,115,188	15	5	4			Whole deletion within LINE1 element	Whole deletion within LINE1 element	0.18	0.259
Chr 16: 88,089,521–88,095,227	5707	2	2			*Alu* element* (*Alu*S)	*Alu* element* (*Alu*S)	0.05	0.5
Chr 19: 35,979,321–35,981,593	2273	6	12			*Alu* element (*Alu*S)	*Alu* element* (*Alu*J)	0.32 (0.88)	1.0E-4
Chr 22: 32,085,572–32,090,063	4492	8	6	*LARGE*	Intron 10–11	*Alu* element* (*Alu*Y)	*Alu* element* (*Alu*Y)	0.80	1.0E-4

Column 1: exact genomic position (UCSC March 2006 genome build) of each deletion, determined from sequencing results; Column 2: size of each deletion (bp) and 3 breakpoint insertions; Column 3: number of samples carrying each deletion (≥1 copy), identified by aCGH or imputed using CNVhap; Column 4: number of chromosomes sequenced–samples with homozygous deletions are scored twice, not all deletions missed by aCGH but imputed by CNVhap were sequenced; Column 5: name of genes (if any) overlapped by deletions; Column 6: introns/exons overlapped by deletions; Columns 7 & 8: these show whether the upstream and/or downstream deletion breakpoints are located within/adjacent to any repeat sequence elements. ^*^ indicates *Alu* elements with poly-A tails flanking the breakpoint; Column 9: max r^2^ scores for LD with surrounding SNPs within 50 kb window for each deletion; Column 10: significance values for r^2^ scores. For the deletion on chromosome 19, the analysis was first performed assuming that the reference sample had a copy number of 2 (i.e. homozygous undeleted), and then re-calculated on the basis that the reference was a heterozygous deletion at this loci. The number in brackets refers to the calculation assuming a heterozygous deleted reference.

Based on this analysis, 3 out of 20 deletions showed extensive sequence identity between the two breakpoints, strongly suggesting that NAHR was the mechanism of their formation. In contrast, three other deletions are unlikely to have been produced by homology-dependent mechanisms since small sequence insertions (3 bp, 4 bp and 6 bp) at the junction between the deletion breakpoints were identified in all samples carrying these deletions. Such breakpoint insertions have previously been reported for an appreciable proportion of CNVs [14].

Further analysis showed that 9 out of 20 deletions contain at least one breakpoint located within an *Alu* element, and that 5 additional deletions have breakpoints within long interspersed nuclear element (LINE) repeats (Table 1 and Figure S1). In two of the putative NAHR instances, the extended sequence similarity was due to a pair of *Alu* elements at the breakpoints. In total, *Alu* family elements were found at 13 out of 40 deletion breakpoints. This represents a significant enrichment of *Alus* (p<0.003 from 1000 simulations, see Materials and Methods), which remains the case even when the 3 presumed NAHR events (including 2 *Alu*-*Alu* recombination events) are excluded from the analysis (p<0.043). Intriguingly, the *Alu* sequence ends either directly at the breakpoint or within the region of sequence similarity surrounding the breakpoints in 10 of these 13 cases. Furthermore, in 9 out of 10 such *Alu* elements, it is the poly-A tail which is immediately adjacent to the breakpoint.

### Repeat element analysis

**Figure 2:**
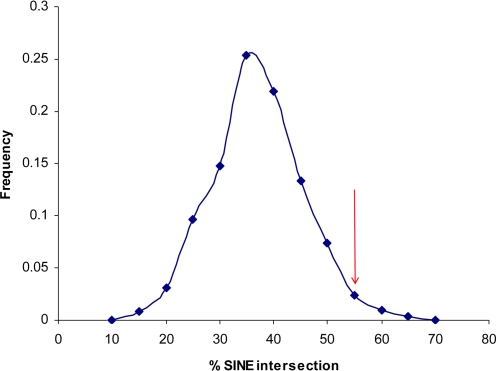
Frequency distribution of the percentage of SINE intersection as computed for 1000 sets of 40 random sequences, compared with the percentage determined for the 40 breakpoint flanking sequences (red arrow).

To investigate the genomic environment in which these CNVs occurred, the sequences flanking each deletion breakpoint (500 bp at each end) were analysed. Initial analysis revealed an over-representation of short interspersed nuclear elements (SINEs), with 22 out of 40 flanking regions intersecting a known SINE element. This was compared with the results of a similar analysis of 1000 random sets of deletions (see Materials and Methods). Figure 2 shows the frequency distribution for the percentage of flanking sequences (from each set of random deletions) that intersected SINE repetitive elements. This distribution is centred at approximately 40% for random sets of 40 sequences, while the 40 deletion flanking sequences derived from our data have 55% intersection with SINE elements. The same percentage (or higher) of intersection was observed in only 37 out of 1000 simulated sets of 40 sequences, yielding an empirical p-value of 0.037. The results of such analyses, however, did not provide support for the enrichment of either segmental duplications or LINEs, nor did it reveal any overlap of segmental duplications in the 500 bp flanking sequences.

**Figure 3:**
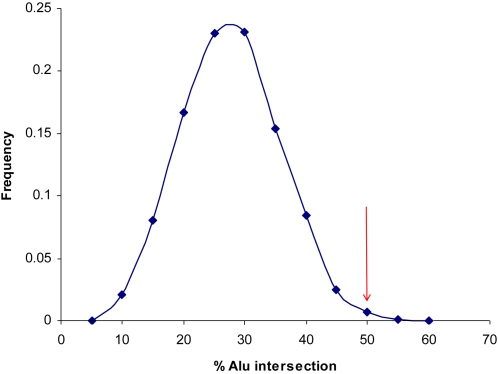
Frequency distribution of the percentage of *Alu* element intersection as computed for 1000 sets of 40 random sequences, compared with the percentage determined for the 40 breakpoint flanking sequences (red arrow).

Further examination of the SINE elements showed that only *Alu* family elements were significantly enriched when compared to the genome average, with 20 of the 40 flanking regions intersecting a known *Alu* element (p<0.008 from 1000 simulations, see Figure 3). Of these, the *Alu*Y subfamily was especially enriched (7 occurrences, p<0.009) compared to the *Alu*S (6 occurrences, p<0.75) and *Alu*J subfamilies (8 occurrences, p<0.019). However, it should be noted that the enrichment of *Alu* elements near deletion breakpoints was less clear (p<0.313) when *Alus* that directly intersected a breakpoint were excluded from the analysis.

Our studies found no evidence for enrichment of repeat elements within the deleted sequences. Excluding *Alu* family elements located at the deletion breakpoints, 7 out of 20 deleted sequences contained at least one *Alu* element (a total of 27 *Alus,* p = 0.789 from 1000 simulations). The distribution of the deleted *Alus* appeared to depend largely on the size of the deletion rather than on the nature of the deletion breakpoints; six out of the seven largest deletions spanned *Alu* elements.

To investigate the association of repeat elements with CNVs on a genome-wide level, further analysis was carried out on all of the variant intervals detected in the original aCGH study from which the 20 deletions were drawn [18]. A significant enrichment of *Alu* elements near CNV breakpoints was observed, together with a small but significant enrichment of LINEs consistent with previous studies which have found an involvement of LINEs in CNV formation [13], [14] (p<0.001 from 1000 simulations in both cases, see Materials and Methods and Figures S2 and S3). It is interesting to note that the enrichment of *Alus* is observed even when the interval between probes is large, under which circumstance one would expect the non-breakpoint sequences to dilute any effect due to enrichment at the breakpoints (as was observed for LINEs). This suggests that, near a significant proportion of CNVs, there is in that region of the genome a general enrichment of *Alus* which extends some distance from the CNV breakpoints.

### Sequence analysis

Initial analysis of the sequences flanking the breakpoints involved searches in 20, 40, 200 and 500 bp flanking sequences (up- and downstream) for regions of >10 bp sequence identity, carried out for 18 out of 20 deletions (excluding the 15 bp and 25 bp deletions for which the flanking sequences overlap). Particularly significant enrichment of regions of sequence similarity was found in the 40 bp analysis, which identified 6 deletions with >10 bp identity–the 3 putative instances of NAHR, 2 pairs of ends involving *Alu* family elements, and homology at the chromosome 1 deletion between an *Alu* poly-A tail and a low complexity AT-rich repeat. For comparison, a similar analysis was performed on 1000 random ensembles of 20 deletions. In all cases there were fewer than 6 pairs with regions of >10 bp sequence identity (i.e. p<10^−3^). Thus, it is clear that similarity between the sequences surrounding the breakpoints plays a role in a proportion of the 20 deletions under study.

Manual inspection of the sequences immediately flanking the deletion breakpoints also suggested that there was stronger sequence similarity than expected at random (Figure S4). After excluding the 3 deletions with extensive sequence identity (presumed to be formed by NAHR) and the 3 deletions with small insertions at the breakpoint junctions (suggesting NHEJ), very short regions of sequence identity (“microhomology”, ≥1 bp) at the breakpoints were observed in 11 out of 14 deletions (79%), as illustrated in Figure 1. By comparison, approximately 56% of randomly assigned breakpoints would be expected to show zero microhomology (75% chance of mismatch at each of the upstream and downstream flanking basepair). Analysis of microhomology at the breakpoints for 1000 random sets of deletions (generated by genome shifts) confirmed this apparent over-representation of microhomology for this set of deletions. This observation applied even to only 1 bp sequence identity at the breakpoints (11/14, p<0.012), but was even stronger for 2 bp (8/14, p<0.001), 3 bp (5/14, p<0.004) and 4 bp (4/14, p<0.002). This enrichment of microhomology was also found when the analysis was repeated using only deletions with *Alus* at their breakpoints, as well as using only deletions with no *Alus* at their breakpoints, indicating that microhomology is not specifically associated with the *Alu* elements. These conclusions remained unchanged when the analysis was extended to include the 3 deletions with an insertion at the breakpoint junction (and which, therefore, showed no microhomology).

**Figure 4:**
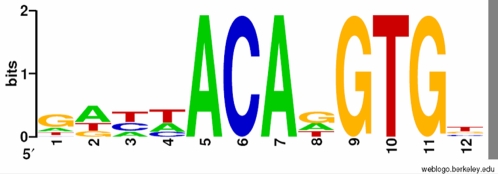
A Shannon logo description of the motif we have found to be enriched around deletion breakpoints. The representation was generated using the WebLogo service (http://weblogo.berkeley.edu/). The x-axis represents the position in the motif, the y-axis represents the certainty we have in the content of that position and the mixture of letters represents the position specific probabilities.

Given the high degree of breakpoint conservation seen in our subset of CNVs, an analysis was carried out to search for possible sequence motifs associated with 19 of the deletions. Analysis using the DRIM software revealed a conserved motif that can be generally described by the IUPAC sequence DHHACADGTG, in 28 out of 38 sequences flanking the breakpoints (p<5×10^−4^) (Figure 4). Of the 28 occurrences of this motif, 10 reside in *Alu* elements, 6 in LINE1 (L1) F Family elements and 4 in other repeat elements.

### Linkage disequilibrium analysis

**Figure 5:**
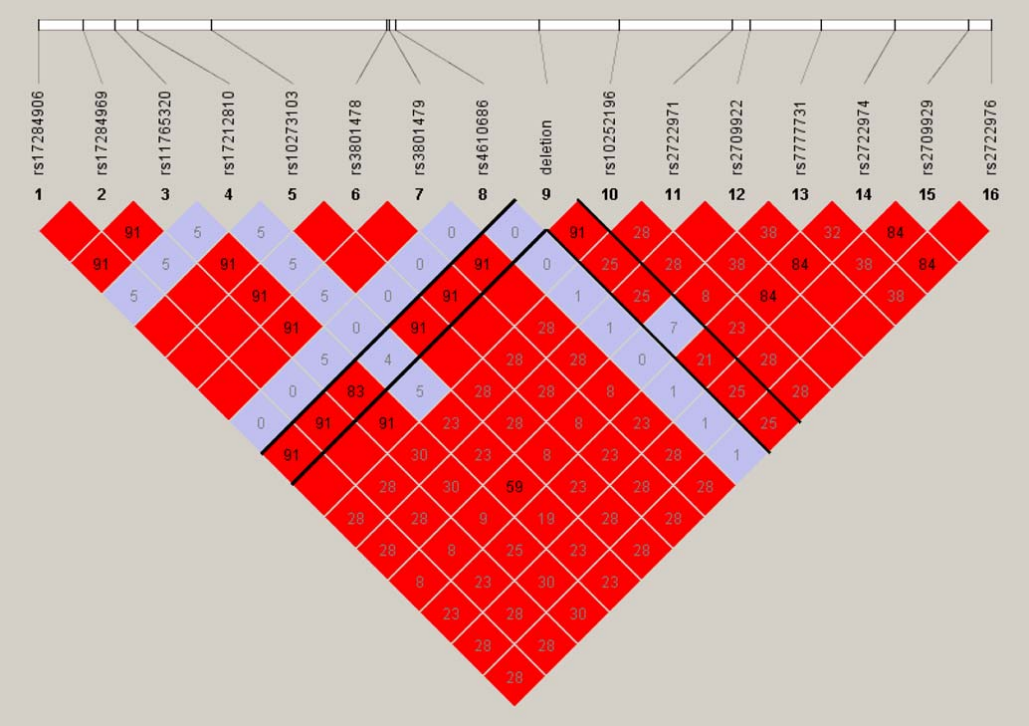
Zoomed in LD plot of deletion on chromosome 7: 82,856,584–82, 857,509, showing the 27 Kb haplotype block containing the deletion. The Haploview default colouring scheme is used: positions are white if LOD <2 and D' <1; blue if LOD <2 and D' = 1; shades of red as a function of D' if LOD ≥2; bright red if D' = 1 and LOD ≥2. Numbers within boxes refer to the r^2^ values between two given positions, so are not directly connected to the colouring scheme. Solid black lines delineate LD between the deletion and other markers in this region. This deletion is in a block of strong LD, and has high LD to all positions in this block (see Figure S5-j for full LD plot).

Linkage disequilibrium (LD) has previously been observed between common deletion CNVs and surrounding SNPs [15], [16]. Therefore, we investigated the extent of LD between the 20 deletions in this study and nearby SNPs genotyped in 35 of the 50 French samples, using Illumina Infinium II 300k arrays. In order to accurately phase confirmed sequence deletions relative to the surrounding SNP genotypes, a recently-developed algorithm was employed (CNVhap, see Materials and Methods). This algorithm was also used to impute genotypes and haplotypic phase for the 35 individuals at HapMap SNPs that are not represented on the Illumina 300K arrays, in order to calculate the LD between the deletions and a very high density SNP panel. An example LD plot is shown in Figure 5 (for further LD plots, see Figure S5). Two measures of LD were investigated; D' measures the degree to which recombination has occurred between the two markers in question, while r^2^ measures the statistical correlation between two loci. All 20 deletions had a D' equal to 1.0 within a 50 kb window either side of the deletion, indicating that no historical recombinations have occurred between the deletion and surrounding markers. Sixteen of the 20 deletions had a maximum r^2^ value, within a 50 kb window either side of the deletion, which was significantly greater (α = 5%) than expected under linkage equilibrium (Table 1). Eight of the 20 deletions had maximum r^2^>0.8, and 11 had r^2^>0.5. This suggests that 8 of the 20 deletions can be tagged with high accuracy by proxy SNPs.

The degree to which the deletions were in Hardy Weinberg equilibrium (HWE) was also investigated (Table S1). All of the deletions were in HWE, except that on chromosome 19 for which all of the deleted samples initially appeared to carry homozygous deletions. Closer investigation of the distribution of aCGH log_2_ intensity ratio scores at this locus (Figure S6) identified a cluster of log_2_ ratios at −5.0, 0.0 and 1.0, which would appear to be consistent with a heterozygous deleted reference sample, so that the cluster at 1.0 corresponds to undeleted samples with a copy number of two. Using CNVhap with the assumption of a single copy reference, deletions and phase were re-inferred in the samples that were not sequenced at this locus, on the basis of aCGH log_2_ ratios and surrounding haplotype structure. Thus, 24 samples were identified as having a single copy deletion, 17 carried homozygous deletions and the remaining 9 were homozygous undeleted. Following this re-analysis, the HWE p-value at this locus was 1.0, indicating that the re-analysed set of deletions is in HWE.

For all 20 deletions, extended haplotypes were identified that are observed in all those with a given deletion (Table S1). These haplotypes include the imputed but untyped Hapmap SNPs. The longest extended haplotype was 234 kb, for the deletion on chromosome 1, and 11 deletions occur in conserved haplotypes of 10 kb or greater. Conserved long range haplotypes indicate that these deletions occurred once and have been inherited together with the surrounding genomic region, as opposed to arising via multiple independent mutation events.

Using the CNVhap algorithm, copy number variation was also imputed in 40 samples that were not initially sequenced as their aCGH scores were below the selection threshold (ADM2, threshold 4). All of these imputed CNV regions were subsequently verified either by sequencing or by viewing the relative log_2_ ratios of samples in the CGH Analytics software (Agilent) (Figure 1). This provides further evidence of the strongly-conserved haplotype structure surrounding these deletions (Figure 5, Figure S5). Three deletions (on chromosomes 2, 5 and 22) actually contain a SNP, and in all cases CNVhap identified that the Illumina log intensity ratios and b-allele frequencies were consistent with a deletion at this SNP in individuals with a confirmed deletion at this locus.

## Discussion

In a recent study, sequencing across the breakpoints of 23 deletions (2–37 kb, median 5836 bp) revealed identical breakpoints across multiple samples, albeit with a maximum of 3 samples sequenced per deletion [14]. The present study represents a more comprehensive survey of a specific subset of small, common CNV deletions (15–5707 bp), with breakpoint sequencing carried out for a mean of 8.2 individuals (10.2 chromosomes) per deletion. Identical CNV breakpoints were found for multiple, unrelated subjects at the sequence level. Together with the common SNP haplotype structure associated with each deletion, these results strongly suggest that the small deletion variants characterised in this study represent ancient events that probably occurred only once and became common in the general population.

The 20 deletions fall into 3 separate classes: three are consistent with being the products of NAHR, in which recombination between stretches of sequence with extensive similarity leads to deletion of the sequence between them; fourteen are simple deletions in which two breakpoints have been rejoined without the involvement of obvious sequence similarity; and three involve small insertions of unknown origin at the site of the breakpoint junction. This is again broadly in line with the recent report [14], which also described these classes of CNV (in respective proportions of 2∶12∶9). The lower proportion of CNVs showing insertions at the breakpoint in the present study may be due to the initial selection criteria which excluded larger deletions–6 of the 9 such insertions in the previous study were associated with deletions larger than 5707 bp (the largest of the 20 deletions in the present study). Alternatively, this may reflect differences in CNV identification bias between the particular custom arrays used in the two studies. Similarly, the high frequency with which deletions were found in or near genes (Table 1) is likely to reflect the fact that the initial identification of CNVs was carried out using a gene-centric CGH-array [18].

The finding, in both the recent deletion sequencing study and in our study, that all copies of CNVs at these locations have an apparently unique origin, contrasts with reports of variable CNV breakpoints, whether in CNVs associated with segmental duplications [4] or arising *de novo* in regions of genomic instability as reported for CNVs associated with autism [8]. This may, to some extent, reflect bias in the selection of CNVs sequenced in these studies–for instance, simple deletions are likely to be more amenable to amplification by PCR than the more complex rearrangements observed in some segmental duplication regions. Nevertheless, it is clear that there are multiple types of CNV region: those containing unique CNVs with invariant breakpoints that are presumed to be derived from single ancient events; regions in which there are multiple, overlapping CNVs with different breakpoints and histories; and unstable regions in which new CNVs are observed to arise on multiple independent occasions.

It is also becoming clear that there are multiple mechanisms of CNV formation, even for the small subset of CNVs represented in this study. A minority of sequenced CNVs can be accounted for by NAHR or other homologous recombination events, usually involving repeat elements ([13], [14], this study). The majority, however, involve the joining of sequences without extensive sequence similarity and are, therefore, likely to be due to other mechanisms.

Repeat sequences such as *Alu* elements are reported to give rise to genomic deletions by promoting recombinational instability [20]. In addition, a sequence comparison between the human and chimpanzee genomes revealed that ∼400 kb of the human genome has been deleted due to *Alu*-mediated recombinations since the human-chimpanzee divergence ∼6 million years ago [21]. There is also evidence that *Alu*-mediated recombination has led to over 650 (∼770 kb) lineage-specific deletions in the chimpanzee genome [22]. The presence of repeat elements around CNV boundaries suggests that NAHR plays a role in their mediation. In our subset of 20 deletions, three cases appear to have been formed by NAHR, two of which were due to recombination between *Alu* elements at each breakpoint. However, even when excluding these CNVs from analysis, there remains a significant enrichment of *Alus* at the deletion breakpoints, suggesting that *Alus* also contribute to other mechanisms of formation involved in the majority of our deletions.

NHEJ has been suggested as the most common method of CNV formation [13], [14]. This process involves the double strand breakage of DNA followed by end joining in the absence of extensive sequence homology, and is associated with small insertions at the junction sites [23]. In three out of the 20 deletions there are small insertions at their breakpoints, and no extensive homology, therefore we suggest these have been mediated by NHEJ.

Both NHEJ and a mechanistically-related repair mechanism, known variously as microhomology-mediated recombination or microhomology-mediated end-joining, are associated with very short stretches of sequence identity (a few bp) between the two ends of the breakpoint junctions [24], [25]. Analysis of the remaining deletions (i.e. lacking extensive sequence homology or insertions at the breakpoint junctions) demonstrated that 8 of the 14 had at least 2 bp microhomology at the breakpoint junctions (all but 3 had at least 1 bp), a highly-significant enrichment. We conclude, therefore, that such microhomology-dependent NHEJ processes were involved in the formation of at least some of the deletions. Although the proposed fork stalling and template switching (FoSTeS) mechanism is also associated with microhomology [26], this has so far only been implicated in the formation of duplications rather than deletions, and thus FoSTeS is unlikely to have been responsible for these particular CNVs.

The initial step of both forms of NHEJ is the formation of one or more DNA double-strand breaks, which may arise spontaneously or be induced, for example, by ionizing radiation or errors during DNA replication. The analysis of sequences flanking the 20 deletions reveals a possible source of such double-strand breaks. For 10 of the deletion breakpoints (out of 40) there is an *Alu* sequence that ends precisely at the breakpoint junction, and in 9 of these cases it is the *Alu* poly-A sequence that is immediately adjacent to the breakpoint. It is clear that the *Alu* poly-A either promotes the formation of deletions or is a target for recombination processes initiated elsewhere in the genome, consistent with previous reports implicating *Alu* elements in recombinational instability [20]. Thus, we propose that *Alu* poly-A sequences are particularly vulnerable to single or double-strand breakage, ultimately leading to the formation of these deletions via NHEJ. Indeed, it is possible that the majority of *Alu*-associated deletions are mediated by *Alu* elements through repair processes initiated by double-strand breaks, rather than through the process of NAHR.

*Alu* poly-A tails are transcribed by RNA polymerase III during retrotransposition [27], and several of the elements will also be transcribed as a result of their location within introns. It has been previously suggested that transcription can promote the formation of DNA breaks along the sequence being transcribed [28], providing a possible basis for the involvement of *Alus* in the initiation of NHEJ. Additionally, *Alu* sequences have been implicated in the formation of non-B DNA structures, such as cruciforms, triplexes, and sticky DNA [29], which are believed to promote genomic deletions enriched for microhomology at their breakpoints [30]. Indeed, the general enrichment of *Alus* around CNVs from our original aCGH study might be explained, in part, by their involvement in non-B DNA conformations. Although single strand breaks and/or deletions may occur at the target sites of *Alu* retrotransposition (mediated by the L1 transposase) [31]–[33], there is no evidence that retrotransposition may have played a role in formation of the deletions investigated in this study–all of the *Alu* elements involved are ancestral, in that similar elements are found at identical locations in the syntenous regions of the chimpanzee genome, with no accompanying deletions being observed.

In addition to the enrichment of *Alu* elements and microhomology, a sequence motif was also identified that was enriched around the deletion breakpoints. Although statistically significant, its mechanistic significance is not yet clear–its enrichment may be an artefact arising from its presence within repeat elements, which are themselves enriched. However, it is tempting to speculate that this motif may be associated with external factors, possibly relating to genomic instability, or may in some way promote non-B DNA conformations.

The high levels of LD found with surrounding SNPs in this study, as in others [15], [16], suggest that the deletion variants typified in the cohorts of these studies can possibly be investigated using one type of marker. For association studies of complex disease, therefore, a subset of variant regions, such as the ones studied here, might be assayed using proxy SNPs or proxy CNVs. In addition, the conserved long range haplotypes indicate that these deletions occurred once and have been inherited together with the surrounding genomic region, as opposed to arising via multiple independent mutation events. Lower LD has been reported between SNP markers and variants located within segmental duplication-associated CNVs, suggesting that these variants often represent recurrent mutational events [17]. This variation in LD may reflect a variation in genomic stability that exists between different CNV subgroups, and the investigation of phenotypic associations with more complex CNV regions or with rare CNVs may require the use of specific detection platforms.

Extrapolation from our data, therefore, suggests that the majority of small, common deletion polymorphisms typified by this study are ancient mutational events that are in LD with surrounding SNPs, and may often be associated with *Alu* repeat elements at their breakpoints. These *Alus* can mediate deletions through NAHR, but we have also shown that they may be involved in double strand breakage leading to NHEJ-associated mechanisms. It is clear from this study, and others, that there are different subtypes of CNV that may arise by different mechanisms. Furthermore, even CNVs that appear to be of the same subtype, such as the small, common deletions investigated in this study, are likely to have different origins. Addressing these issues will require the collection of a more extensive set of flanking sequences.

Two recent studies [13], [34] have also included investigation of the presence of repeat elements at CNV breakpoints. Both confirmed the association of CNV breakpoints with segmental duplications, as previously reported [11]. Significant enrichments of L1 elements [13] and microsatellites were also found [34] but, intriguingly, neither study found a significant association with *Alu* repeats. This is in direct contrast to the clear enrichment of *Alu* repeats found at the flanking regions of deletion breakpoints in the present study, and to the absence of evidence for an association with either L1 repeats or segmental duplications. In addition, analysis of the entire set of aCGH data from our previous study [18] has also revealed a significant enrichment of *Alus* (and also LINE repeats) around the breakpoints of CNVs, confirming that these repeats might play a role in the formation of some CNVs. This is further supported by a previous analysis of the CNVs documented on the TCAG Database of Genomic Variants [32], which showed the distribution of CNVs in the genome to be correlated with *Alu* repeats [12]. Our aCGH data was derived from high-resolution custom arrays, and was therefore more likely to identify the smaller deletions which appear to be particularly associated with *Alu* elements. We consider it likely that this apparent discrepancy has arisen because of biases inherent in different CNV identification platforms. Thus, not only are there different types of CNV and different mechanisms of their formation, but the different sub-types may also have different size distributions. Future studies attempting global analysis of CNVs will need to take account of this growing CNV diversity.

## Materials and Methods

### PCR

The 20 deletion variants investigated were a subset of CNVs that met the following criteria in our previous study: deleted compared to the reference sample, called in ≥2/50 samples, common boundaries, and estimated size <3 kb as predicted from the aCGH data (although the aCGH algorithm underestimated the size of some aberrations, thus 4 deletions were later found to be >3 kb). These criteria gave 55 small, common deletions in total. Fourteen primer pairs failed to amplify and 21 showed no obvious size change on agarose gel electrophoresis; these deletions were not investigated further. Individual frequencies of the remaining 20 variants were between 4% and 88%, and mean predicted size was 1373 bp. The high-resolution of aCGH tools used in our initial CNV screen [18] allowed precise mapping of boundaries. Breakpoint regions were amplified by PCR, using primer pairs designed to flank the position of the deletions as predicted by the genomic locations of the aCGH probes. Primers were designed using Primer3 software [35] (sequences available on request). Long-range PCR was performed, using a Qiagen LongRange PCR Kit (see manufacturers protocol for details). All PCR products were examined by agarose gel electrophoresis. Products were purified using the GenElute^TM^ PCR Clean-Up Kit (Sigma-Aldrich), which uses a series of washes and spins to purify the DNA. The concentration of each purified PCR product was then quantitated using a Nanodrop ND-1000 spectrophotometer, to calculate the amount of PCR product required for sequencing.

### DNA sequencing

DNA sequencing was carried out using an ABI 3730xl DNA Analyzer (Applied Biosystems), with purified PCR products for each deletion sequenced in both the forward and reverse direction using the corresponding PCR primers. Sequence files were visualised using the Chromas 2.01 software (Technelysium Pty Ltd.). The UCSC Genome Browser [19] was used to retrieve the reference genome sequence (March 2006 build), with forward and reverse PCR primers used to calculate the start and end points of the sequence for each region. Multiple alignments were then carried out between the deleted sample sequences and the corresponding reference genome sequence, using the ClustalW programme [36]. The upstream sequence breakpoints for each deletion were determined by aligning the reference sequence with the sequences generated using the forward primers in the sequencing reaction. Downstream breakpoints were determined by aligning the reference sequence with the reverse complements of the sequences generated using the reverse primers.

### Repeat element analysis

Initial identification of repeat sequence elements at the deletion breakpoints was carried out using RepeatMasker [37]. For the investigation of sequences flanking each deletion, sequences 500 bp upstream of the deletion start and 500 bp downstream of the deletion end were considered. Repetitive elements annotation was taken from the RepeatMasker track in the UCSC Table Browser [38] (March 2006). For each flanking sequence, we determined whether it intersects a repetitive element, and the percentage of sequences with an intersection was computed amongst all 40 flanking sequences. To determine an empirical p-value for the observed percentage of intersection we used the following simulations procedure: a new set of “test” deletions was generated, for each observed deletion, by randomly selecting a stretch of sequence of the same length and on the same chromosome as the original; the number of this set of random sequences that intersected a repetitive element was then counted as above. This procedure was repeated 1000 times to derive an empirical p-value for the enriched intersection we observe for the flanking sequences in the actual data. The same analysis was performed separately for *Alu* elements only, and for LINE1 elements only.

For further analysis of *Alu* intersection with breakpoints and presence within deleted sequences, a similar strategy was adopted using EnsEMBL release 47 (Oct 2007) accessed via its Perl API [39]. Random sets of deletions were generated (again with the same lengths and on the same chromosomes as the original set), and each deletion was scored for the presence of *Alus* intersecting/between the breakpoints. Empirical p-values were determined by comparison of the resulting scores with the experimental data.

Genome-wide analysis of the copy number variable regions identified in the original CGH study [18] was carried out as follows. After converting the probe list from the 244K feature Agilent microarray to the latest genome build (NCBI 36.2), 15,684 regions were identified which, for at least one individual, lay between a probe previously scored as copy number variable and a probe which was unchanged compared to the reference sample–i.e. all those regions were identified which harbour CNV boundaries. The number of repeat elements (*Alu* or LINE) present within each inter-probe region was determined (according to EnsEMBL build 47), and each region assigned a score for element density (number of elements per kb). To provide a statistical comparison for this analysis, 1000 random sets of the 15,684 probe intervals were assembled. For each set of intervals (observed CNV breakpoint regions and random sets), inter-probe regions were ordered according to sequence length, and cumulative plots were constructed of mean element density versus mean interval length.

### Sequence motif analysis

For 19 of the validated deletions, two sequences were extracted, 1000 bp upstream of the deletion start and 1000 bp downstream from the deletion end. The DRIM software package [40] was used to search for over-representation of sequence motifs in this set of sequences. DRIM exhaustively searches through a predefined motif space, and using a hyper-geometric distribution, finds those motifs that have higher occurrence rate in the target set compared to a background sequence set. 1000 sequences, 1000 bp long each, from random locations in the genome were chosen to represent the background. After a motif is found to be enriched, DRIM applies an expansion heuristic to optimize it to the target set. For further details on the heuristic expansion see Eden *et al*. [40]. The resulting hyper-geometric p-value is corrected for the size of the initial exhaustively searched motif space. An exact comparison of the positions of the breakpoints, of the motif occurrences and of their relationship to repeat elements can be viewed by using the supplemental custom track files in the UCSC Repeat Masker Track, shown in Tables S2 (flanking sequences) and S3 (motif), and exemplified in Figures S7 and S8.

Further analysis was carried out to determine how frequently one would observe an enriched sequence motif if random genomic sequences are used as input for the DRIM algorithm. The DRIM algorithm was run 10 times on random data generated in the following way: i) 1000 1 kb sequences were drawn randomly on the genome, to serve as a background as in the motif search in our actual data; ii) For each of the 10 instances, a set of 19 deletions were drawn by randomly shifting the actual detected deletions along their chromosomes. The best corrected hypergeometric enrichment observed was p = ∼0.5. Given that we ran the process 10 times, this is expected, and confirms that our result of p = 10^−4^ after correction indicates a significant enrichment of our discovered motif.

### Sequence similarity analysis

Flanking sequences of 20, 40, 200 and 500 bp for 18/20 deletions (minus the 2 deletions <25 bp) were analysed for stretches of sequence identity. In addition, sequence similarity searches were carried out between the regions 30 bp downstream of the left breakpoint and 30 bp upstream of the right breakpoint, and vice-versa. Each deletion was scored according to the length of the maximum stretch of sequence identity. The analysis was then repeated for 1000 random ensembles of 18 sequence pairs obtained by shifting the breakpoints of the 18 deletions in tandem along their chromosome. P-values were determined by analysing the frequency with which stretches of sequence identity >10 bp were identified for the actual deletions compared to the 1000 random ensembles.

Microhomology analysis was carried out in a similar manner to repeat element analysis (see above), again using the EnsEMBL Perl API to access sequence data. For the particular subset of deletions under study, 1000 sets of random deletions were generated with the same characteristics. 20 bp sequences spanning the pairs of breakpoints were compared, aligning the breakpoints with each other, and the extent of 100% sequence identity flanking/spanning the breakpoints was determined. These simulations were then scored against the original data to give empirical p-values. Figure S4 shows sequence alignments between the breakpoints for each of the 20 deletions, illustrating the scoring of microhomology.

### Linkage disequilibrium analysis

This analysis used data from SNP genotyping of 35 of the 50 samples, carried out using Illumina Infinium II 300K arrays. For the accurate phasing of confirmed sequence deletions relative to surrounding SNP genotypes, a novel algorithm CNVhap was developed (manuscript in preparation). This algorithm is an extension of the fastPHASE algorithm [41] to accommodate CNVs as well as SNPs, and to directly model the Illumina b-allele frequency and log intensity ratios, as well as the aCGH log intensity ratios. CNVhap also imputes genotypes at untyped Hapmap SNPs, as well as imputing deletions in samples, based on the conservation of haplotype structure. LD calculations and plots were conducted in Haploview [42]. Significance values for r^2^ were calculated by permuting the alleles at the deletion 10,000 times; we note that, in order to achieve a high r^2^, the minor allele frequency of the deletion and the surrounding marker must be similar, which was not always the case. Haplotype blocks were identified using the 4 gamete test as implemented in Haploview.

## Supporting Information

Figure S1 Output information from RepeatMasker programme for each of the 20 deletions. This gives a summary table of repeat sequences (1), including the types and lengths of repeat elements present in each sequence, an annotation file showing the position and order of these elements within the sequence (2), and a masked file of the reference sequence to again show where these elements are present (basepairs within repeat sequence masked by ‘N’) (3). The order of deletions is the same as shown in Table 1: a) Chr 1: 145,312,298–145,314,875; b) Chr 2: 229,467,533–229,468,151; c) Chr 3: 181,137,036–181,137,500; d) Chr 4: 98,573,315–98,578,237; e) Chr 5: 65,479,440–65,479,975; f) Chr 5: 78,145,556–78,147,626; g) Chr 6: 24,433,346–24,435,791; h) Chr 6: 34,425,089–34,427,582; i) Chr 6: 162,645,085–162,645,903; j) Chr 7: 82,856,584–82,857,509; k) Chr 12: 20,859,912–20,859,936; l) Chr 14: 72,402,707–72,403,561; m) Chr 14: 72,615,524–72,616,685; n) Chr 15: 83,858,016–83,860,206; o) Chr 16: 22,955,277–22,957,032; p) Chr 16: 56,282,301–56,285,908; q) Chr 16: 76,115,174–76,115,188; r) Chr 16: 88,089,521–88,095,227; s) Chr 19: 35,979,321–35,981,593; t) Chr 22: 32,085,572–32,090,063.
(0.03 MB ZIP)

Figure S2 Cumulative plot of Alu element density as a function of the mean inter-probe interval. All probe intervals harbouring a CNV breakpoint were ranked according to size and scored according to the number of Alu elements intersecting that interval. Alu density was determined as the cumulative total of the number of elements divided by the total interval length. Dotted lines show the 95% confidence intervals determined using 1000 randomly selected sets of probe intervals. Note that Alu elements are not scored in appreciable numbers until the interval length reaches ∼300 bp (mean interval ∼180 bp), due to the microarray being designed so as to avoid placing probes within repetitive elements.
(0.27 MB TIF)

Figure S3 As for Figure S2, for LINE elements.
(0.28 MB TIF)

Figure S4 DNA sequences at breakpoint junctions. Reference genome sequences spanning each breakpoint junction are shown aligned against each other according to the sequence present on chromosomes carrying deletions (highlighted in red). * indicates blocks of sequence identity/microhomology at the breakpoint junctions; + indicates further stretches of sequence similarity surrounding some breakpoint junctions.
(0.03 MB DOC)

Figure S5 Plots show linkage disequilibrium (LD) of SNPs within 100 kb of each deletion. The default colouring scheme of Haploview is used, whereby positions are coloured white if LOD <2 and D' <1; blue if LOD <2 and D' = 1; shades of red as a function of D' if LOD≥2; bright red if D' = 1 and LOD ≥2. Numbers within the box refer to the r2 values between two given positions, and so are not directly connected to the colouring scheme. The solid black lines delineate the LD between the deletion and other markers in this region. a) Deletion at chr 1: 145,312,298–145,314,875; b) Deletion at Chr 2: 229,467,533–229,468,151; c) Deletion at Chr 3: 181,137,036–181,137,500; d) Deletion at Chr 4: 98,573,315–98,578,237; e) Deletion at Chr 5: 65,479,440–65,479,975; f) Deletion at Chr 5: 78,145,556–78,147,626; g) Deletion at Chr 6: 24,433,346–24,435,791; h) Deletion at Chr 6: 34,425,089–34,427,582; i) Deletion at Chr 6: 162,645,085–162,645,903; j) Deletion at Chr 7: 82,856,584–82,857,509; k) Deletion at Chr 12: 20,859,912–20,859,936; l) Deletion at Chr 14: 72,402,707–72,403,561; m) Deletion at Chr 14: 72,615,524–72,616,685; n) Deletion at Chr 15: 83,858,016–83,860,206; o) Deletion at Chr 16: 22,955,277–22,957,032; p) Deletion at Chr 16: 56,282,301–56,285,908; q) Deletion at Chr 16: 76,115,174–76,115,188; r) Deletion at Chr 16: 88,089,521–88,095,227; s) Deletion at Chr 19: 35,979,321–35,981,593; t) Deletion at Chr 22: 32,085,572–32,090,063.
(8.90 MB ZIP)

Figure S6 Histogram of Agilent aCGH log2 intensity ratios at probe located at chr 19: 35,979,761–35,979,820. With the assumption of a single copy deletion reference, the scores <2 are taken to be homozygous deletions; those with scores ∼0.0 are taken to be heterozygous deletions, and those with scores ∼1.0 are taken to be normal two copy samples.
(0.09 MB TIF)

Figure S7 Example of UCSC Genome Browser representation of the genomic environment of the mapped breakpoints, showing motif sequence occurring in both flanking sequences of deletion. Using the custom track .bed files in Supplementary Tables 2 and 3, one can view 1 kb sequences flanking the breakpoints (green bars) together with the occurrences of the motif DHHACADGTG (red bars) and the repeat elements tracks (standard UCSC tracks). To generate the two custom tracks for this visual representation of the data, a user of the UCSC Genome browser would go to “manage custom tracks” (just under the genome view) and submit the .bed files. The repeat elements tracks need to also be turned on.
(3.22 MB TIF)

Figure S8 Same as Figure S7, but the motif can be seen to co-occur in repeat elements as well as in the flanking sequences on both sides.
(3.24 MB TIF)

Table S1 Identification of extended deletion haplotypes and Hardy-Weinberg equilibrium. For each deletion, we identified the longest extended haplotype which was common to 100% of haplotypes with this deletion. We report the haplotype, the length of the haplotype. For the deletion on chromosome 19, we report the results of the calculation assuming a reference with 2 copies as well as the results assuming a reference with 1 copy (in brackets).
(0.04 MB DOC)

Table S2 This is the .bed file for the custom track for the 1 kb sequences flanking the deletion breakpoints (green bars), to be uploaded into UCSC Genome Browser.
(0.01 MB XLS)

Table S3 This is the .bed file for the custom track for the motif occurrences (red bars), to be uploaded into UCSC Genome Browser.
(0.02 MB XLS)

## References

[1] Iafrate AJ, Feuk L, Rivera MN, Listewnik ML, Donahoe PK (2004). Detection of large-scale variation in the human genome.. Nat Genet.

[2] Sebat J, Lakshmi B, Troge J, Alexander J, Young J (2004). Large-scale copy number polymorphism in the human genome.. Science.

[3] Tuzun E, Sharp AJ, Bailey JA, Kaul R, Morrison VA (2005). Fine-scale structural variation of the human genome.. Nat Genet.

[4] Redon R, Ishikawa S, Fitch KR, Feuk L, Perry GH (2006). Global variation in copy number in the human genome.. Nature.

[5] Wong KK, deLeeuw RJ, Dosanjh NS, Kimm LR, Cheng Z (2007). A comprehensive analysis of common copy-number variations in the human genome.. Am J Hum Genet.

[6] Database of Genomic Variants–A curated catalogue of structural variation in the human genome.. http://projects.tcag.ca/variation/.

[7] Stranger BE, Forrest MS, Dunning M, Ingle CE, Beazley C (2007). Relative impact of nucleotide and copy number variation on gene expression phenotypes.. Science.

[8] Sebat J, Lakshmi B, Malhotra D, Troge J, Lese-Martin C (2007). Strong association of de novo copy number mutations with autism.. Science.

[9] Fanciulli M, Norsworthy PJ, Petretto E, Dong R, Harper L (2007). FCGR3B copy number variation is associated with susceptibility to systemic, but not organ-specific, autoimmunity.. Nat Genet.

[10] Gonzalez E, Kulkarni H, Bolivar H, Mangano A, Sanchez R (2005). The influence of CCL3L1 gene-containing segmental duplications on HIV-1/AIDS susceptibility.. Science.

[11] Sharp AJ, Locke DP, McGrath SD, Cheng Z, Bailey JA (2005). Segmental duplications and copy-number variation in the human genome.. Am J Hum Genet.

[12] Cooper GM, Nickerson DA, Eichler EE (2007). Mutational and selective effects on copy-number variants in the human genome.. Nat Genet.

[13] Korbel JO, Urban AE, Affourtit JP, Godwin B, Grubert F (2007). Paired-end mapping reveals extensive structural variation in the human genome.. Science.

[14] Perry GH, Ben-Dor A, Tsalenko A, Sampas N, Rodriguez-Revenga L (2008). The fine-scale and complex architecture of human copy-number variation.. Am J Hum Genet.

[15] Hinds DA, Kloek AP, Jen M, Chen X, Frazer KA (2006). Common deletions and SNPs are in linkage disequilibrium in the human genome.. Nat Genet.

[16] McCarroll SA, Hadnott TN, Perry GH, Sabeti PC, Zody MC (2006). Common deletion polymorphisms in the human genome.. Nat Genet.

[17] Locke DP, Sharp AJ, McCarroll SA, McGrath SD, Newman TL (2006). Linkage disequilibrium and heritability of copy-number polymorphisms within duplicated regions of the human genome.. Am J Hum Genet.

[18] de Smith AJ, Tsalenko A, Sampas N, Scheffer A, Yamada NA (2007). Array CGH analysis of copy number variation identifies 1284 new genes variant in healthy white males: implications for association studies of complex diseases.. Hum Mol Genet.

[19] Karolchik D, Baertsch R, Diekhans M, Furey TS, Hinrichs A (2003). The UCSC Genome Browser Database.. Nucleic Acids Res.

[20] Krawczak M, Cooper DN (1991). Gene deletions causing human genetic disease: mechanisms of mutagenesis and the role of the local DNA sequence environment.. Hum Genet.

[21] Sen SK, Han K, Wang J, Lee J, Wang H (2006). Human genomic deletions mediated by recombination between Alu elements.. Am J Hum Genet.

[22] Han K, Lee J, Meyer TJ, Wang J, Sen SK (2007). Alu recombination-mediated structural deletions in the chimpanzee genome.. PLoS Genet.

[23] Linardopoulou EV, Williams EM, Fan Y, Friedman C, Young JM (2005). Human subtelomeres are hot spots of interchromosomal recombination and segmental duplication.. Nature.

[24] Chan CY, Kiechle M, Manivasakam P, Schiestl RH (2007). Ionizing radiation and restriction enzymes induce microhomology-mediated illegitimate recombination in Saccharomyces cerevisiae.. Nucleic Acids Res.

[25] Lee K, Lee SE (2007). Saccharomyces cerevisiae Sae2- and Tel1-dependent single-strand DNA formation at DNA break promotes microhomology-mediated end joining.. Genetics.

[26] Lee JA, Carvalho CM, Lupski JR (2007). A DNA replication mechanism for generating nonrecurrent rearrangements associated with genomic disorders.. Cell.

[27] Dewannieux M, Heidmann T (2005). Role of poly(A) tail length in Alu retrotransposition.. Genomics.

[28] Gonzalez-Barrera S, Garcia-Rubio M, Aguilera A (2002). Transcription and double-strand breaks induce similar mitotic recombination events in Saccharomyces cerevisiae.. Genetics.

[29] Bacolla A, Jaworski A, Larson JE, Jakupciak JP, Chuzhanova N (2004). Breakpoints of gross deletions coincide with non-B DNA conformations.. Proc Natl Acad Sci U S A.

[30] Wells RD (2007). Non-B DNA conformations, mutagenesis and disease.. Trends Biochem Sci.

[31] Callinan PA, Wang J, Herke SW, Garber RK, Liang P (2005). Alu retrotransposition-mediated deletion.. J Mol Biol.

[32] Cost GJ, Boeke JD (1998). Targeting of human retrotransposon integration is directed by the specificity of the L1 endonuclease for regions of unusual DNA structure.. Biochemistry.

[33] Dewannieux M, Esnault C, Heidmann T (2003). LINE-mediated retrotransposition of marked Alu sequences.. Nat Genet.

[34] Kim PM, Korbel JO, Chen X, Gerstein MB (2007). Copy Number Variants and Segmental Duplications Show Different Formation Signatures.. http://arxiv.org/abs/0709.4200v1.

[35] Rozen S, Skaletsky H (2000). Primer3 on the WWW for general users and for biologist programmers.. Methods Mol Biol.

[36] Chenna R, Sugawara H, Koike T, Lopez R, Gibson TJ (2003). Multiple sequence alignment with the Clustal series of programs.. Nucleic Acids Res.

[37] Smit AFA, Hubley R, Green P (1996–2004). RepeatMasker Open-3.0.. http://www.repeatmasker.org.

[38] Karolchik D, Hinrichs AS, Furey TS, Roskin KM, Sugnet CW (2004). The UCSC Table Browser data retrieval tool.. Nucleic Acids Res.

[39] Hubbard TJ, Aken BL, Beal K, Ballester B, Caccamo M (2007). Ensembl 2007.. Nucleic Acids Res.

[40] Eden E, Lipson D, Yogev S, Yakhini Z (2007). Discovering motifs in ranked lists of DNA sequences.. PLoS Comput Biol.

[41] Scheet P, Stephens M (2006). A fast and flexible statistical model for large-scale population genotype data: applications to inferring missing genotypes and haplotypic phase.. Am J Hum Genet.

[42] Barrett JC, Fry B, Maller J, Daly MJ (2005). Haploview: analysis and visualization of LD and haplotype maps.. Bioinformatics.

