# Supplementary figures and images for: Small Deletion Variants Have Stable Breakpoints Commonly Associated with Alu Elements
Source: PLoS One. 2008 Aug 29;3(8):e3104. doi: 10.1371/journal.pone.0003104 (PMC2518860; doi:10.1371/journal.pone.0003104)

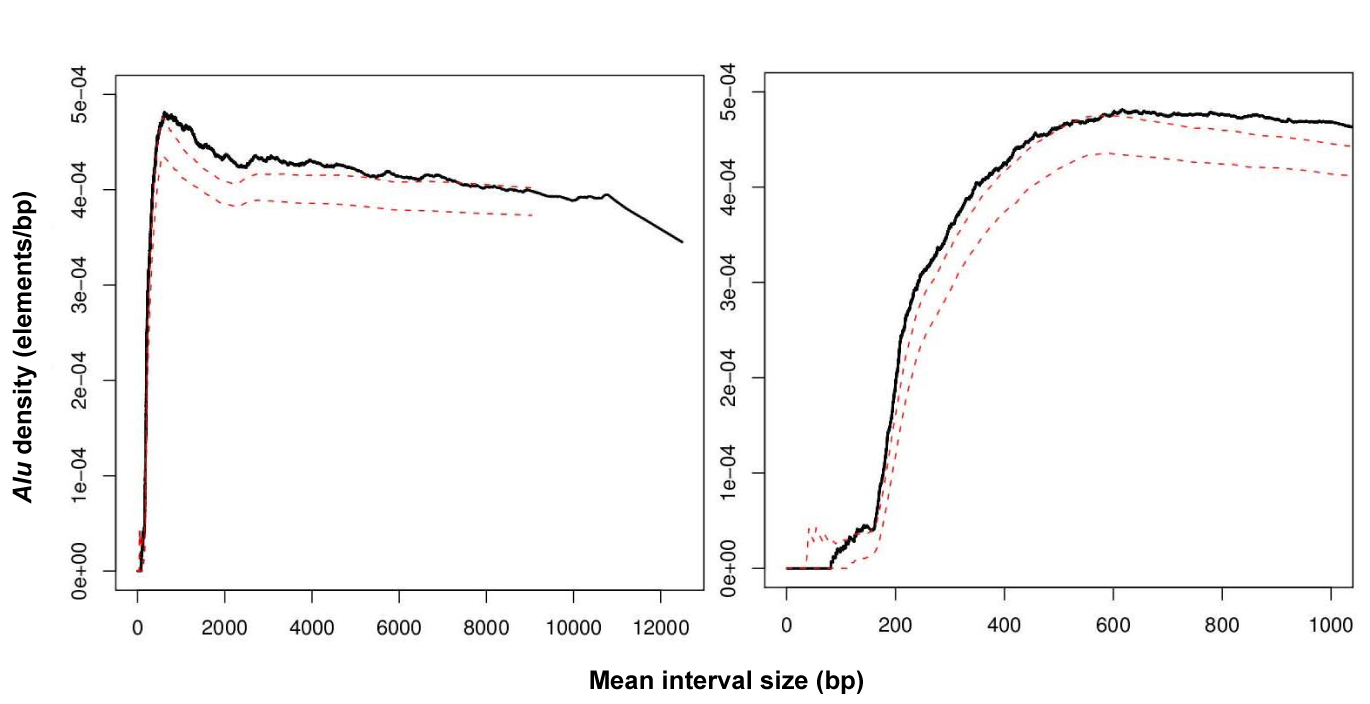

Supplement: Figure S2 — Cumulative plot of Alu element density as a function of the mean inter-probe interval. All probe intervals harbouring a CNV breakpoint were ranked according to size and scored according to the number of Alu elements intersecting that interval. Alu density was determined as the cumulative total of the number of elements divided by the total interval length. Dotted lines show the 95% confidence intervals determined using 1000 randomly selected sets of probe intervals. Note that Alu elements are not scored in appreciable numbers until the interval length reaches ∼300 bp (mean interval ∼180 bp), due to the microarray being designed so as to avoid placing probes within repetitive elements. (0.27 MB TIF) [file pone.0003104.s002.tif]

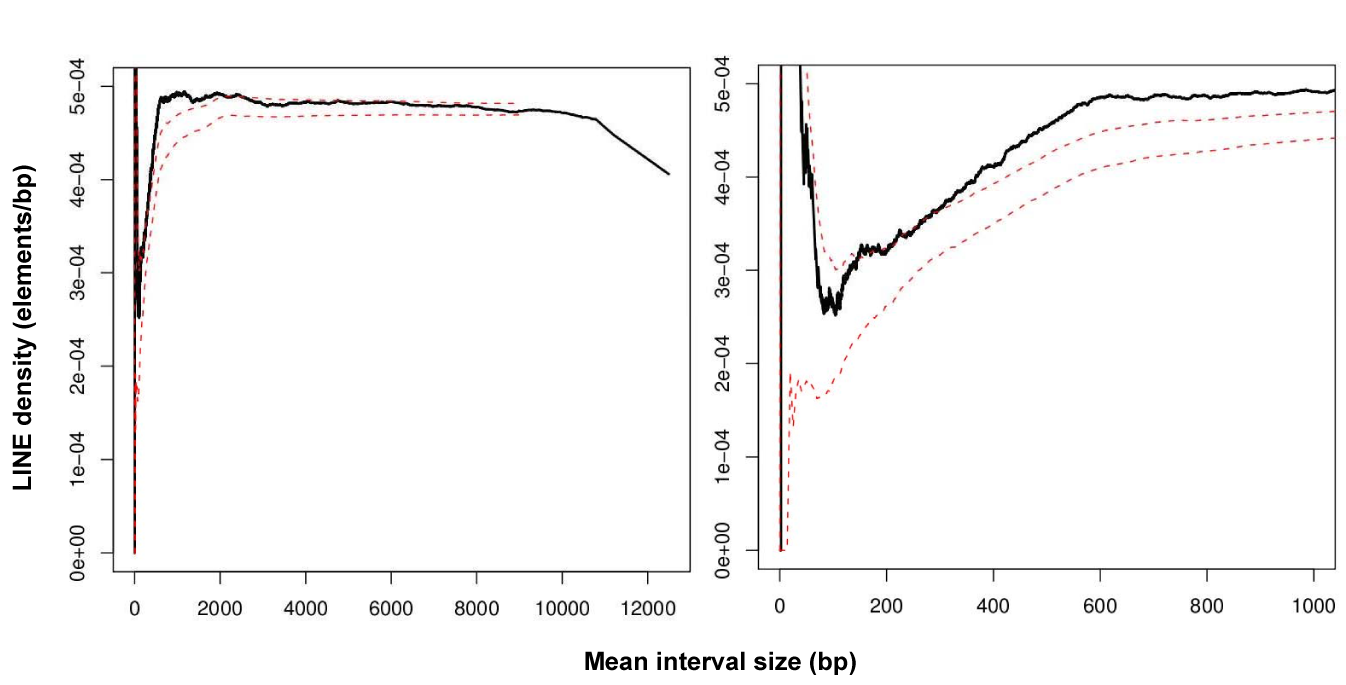

Supplement: Figure S3 — As for Figure S2, for LINE elements. (0.28 MB TIF) [file pone.0003104.s003.tif]

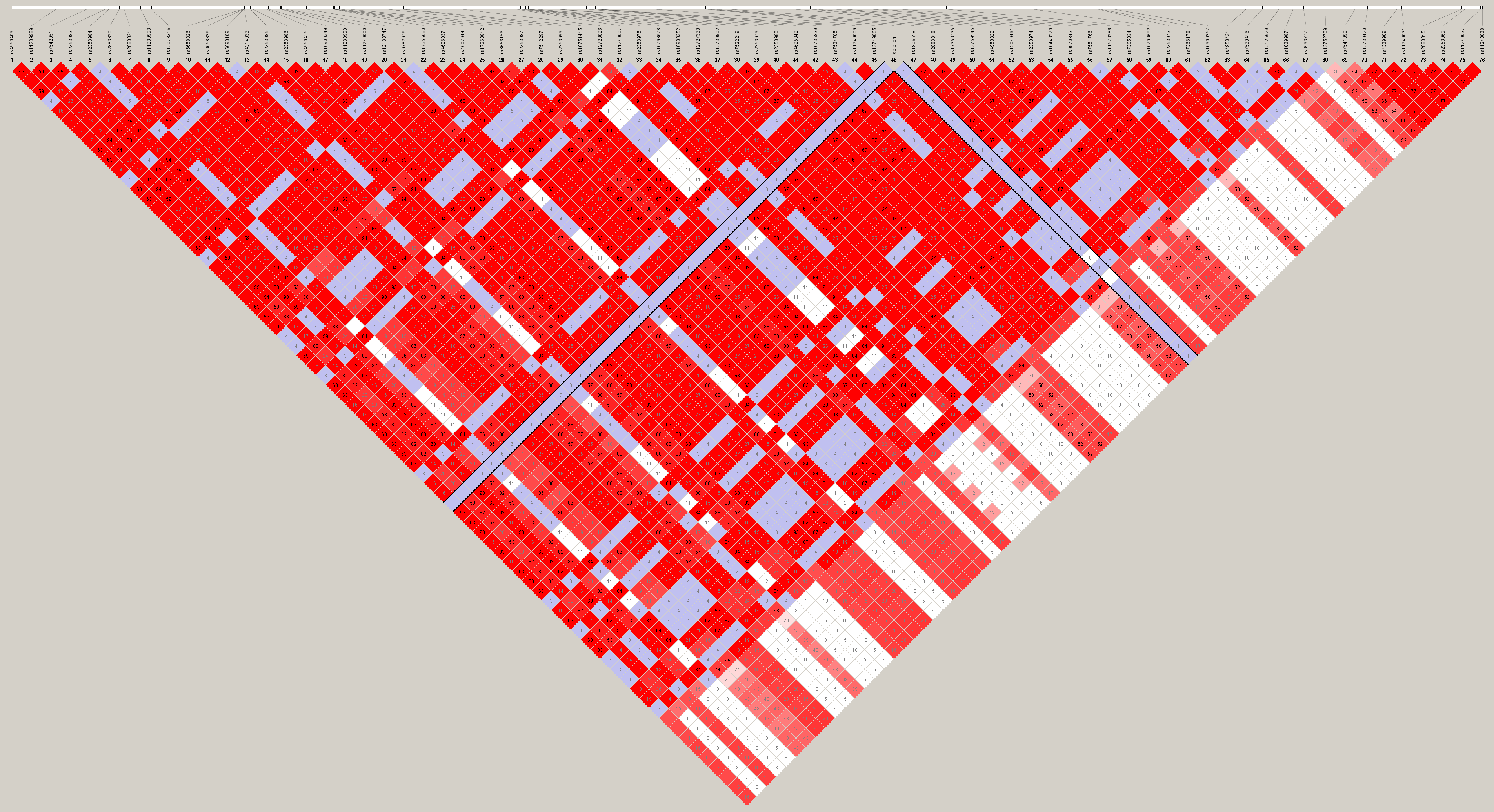

Supplement: Figure S5 — Plots show linkage disequilibrium (LD) of SNPs within 100 kb of each deletion. The default colouring scheme of Haploview is used, whereby positions are coloured white if LOD <2 and D' <1; blue if LOD <2 and D' = 1; shades of red as a function of D' if LOD≥2; bright red if D' = 1 and LOD ≥2. Numbers within the box refer to the r2 values between two given positions, and so are not directly connected to the colouring scheme. The solid black lines delineate the LD between the deletion and other markers in this region. a) Deletion at chr 1: 145,312,298–145,314,875; b) Deletion at Chr 2: 229,467,533–229,468,151; c) Deletion at Chr 3: 181,137,036–181,137,500; d) Deletion at Chr 4: 98,573,315–98,578,237; e) Deletion at Chr 5: 65,479,440–65,479,975; f) Deletion at Chr 5: 78,145,556–78,147,626; g) Deletion at Chr 6: 24,433,346–24,435,791; h) Deletion at Chr 6: 34,425,089–34,427,582; i) Deletion at Chr 6: 162,645,085–162,645,903; j) Deletion at Chr 7: 82,856,584–82,857,509; k) Deletion at Chr 12: 20,859,912–20,859,936; l) Deletion at Chr 14: 72,402,707–72,403,561; m) Deletion at Chr 14: 72,615,524–72,616,685; n) Deletion at Chr 15: 83,858,016–83,860,206; o) Deletion at Chr 16: 22,955,277–22,957,032; p) Deletion at Chr 16: 56,282,301–56,285,908; q) Deletion at Chr 16: 76,115,174–76,115,188; r) Deletion at Chr 16: 88,089,521–88,095,227; s) Deletion at Chr 19: 35,979,321–35,981,593; t) Deletion at Chr 22: 32,085,572–32,090,063. (8.90 MB ZIP) [file pone.0003104.s005.zip › SuppFig5/1_145312298_145314875_res.emphase.LD.PNG]

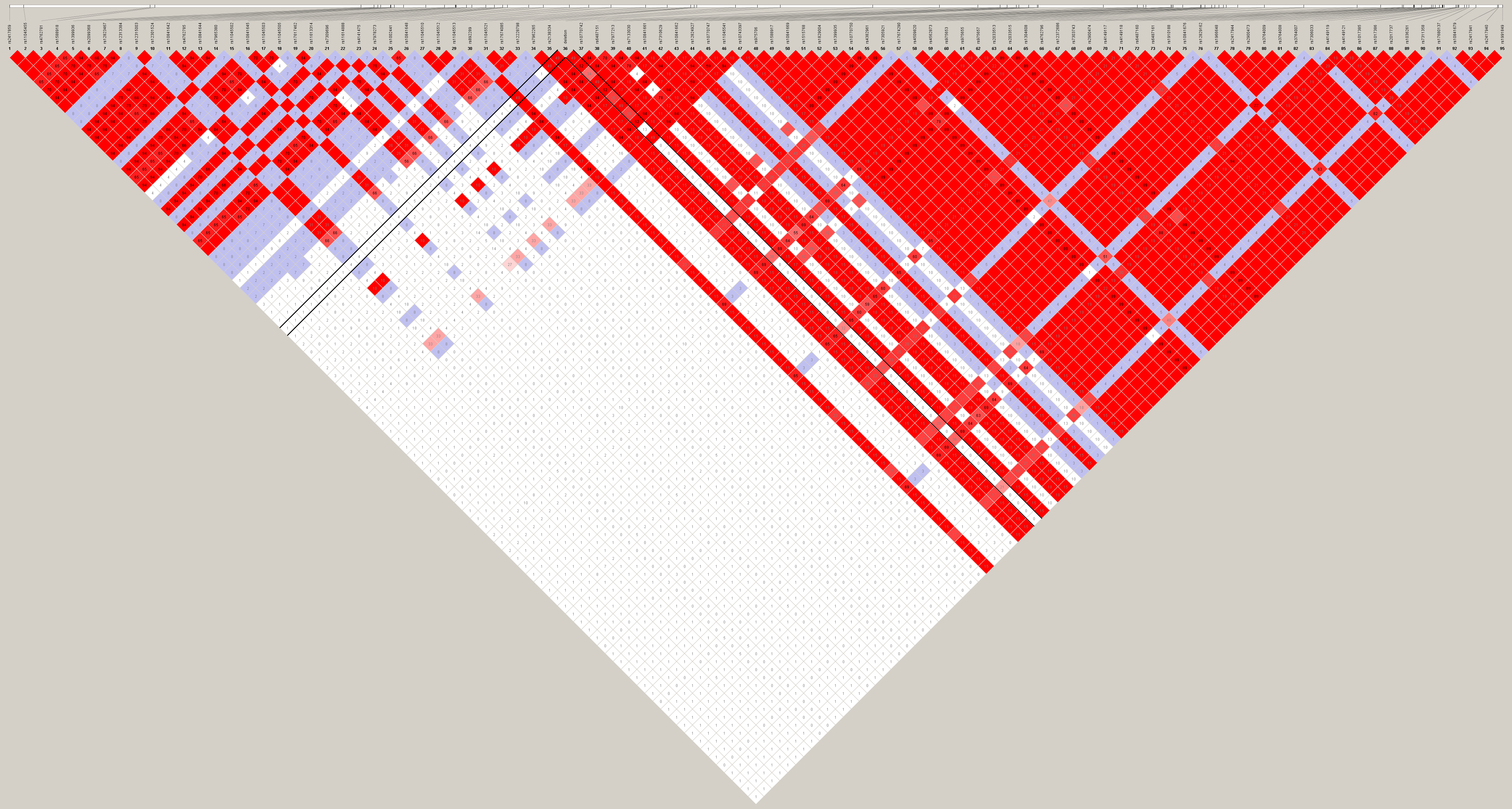

Supplement: Figure S5 — Plots show linkage disequilibrium (LD) of SNPs within 100 kb of each deletion. The default colouring scheme of Haploview is used, whereby positions are coloured white if LOD <2 and D' <1; blue if LOD <2 and D' = 1; shades of red as a function of D' if LOD≥2; bright red if D' = 1 and LOD ≥2. Numbers within the box refer to the r2 values between two given positions, and so are not directly connected to the colouring scheme. The solid black lines delineate the LD between the deletion and other markers in this region. a) Deletion at chr 1: 145,312,298–145,314,875; b) Deletion at Chr 2: 229,467,533–229,468,151; c) Deletion at Chr 3: 181,137,036–181,137,500; d) Deletion at Chr 4: 98,573,315–98,578,237; e) Deletion at Chr 5: 65,479,440–65,479,975; f) Deletion at Chr 5: 78,145,556–78,147,626; g) Deletion at Chr 6: 24,433,346–24,435,791; h) Deletion at Chr 6: 34,425,089–34,427,582; i) Deletion at Chr 6: 162,645,085–162,645,903; j) Deletion at Chr 7: 82,856,584–82,857,509; k) Deletion at Chr 12: 20,859,912–20,859,936; l) Deletion at Chr 14: 72,402,707–72,403,561; m) Deletion at Chr 14: 72,615,524–72,616,685; n) Deletion at Chr 15: 83,858,016–83,860,206; o) Deletion at Chr 16: 22,955,277–22,957,032; p) Deletion at Chr 16: 56,282,301–56,285,908; q) Deletion at Chr 16: 76,115,174–76,115,188; r) Deletion at Chr 16: 88,089,521–88,095,227; s) Deletion at Chr 19: 35,979,321–35,981,593; t) Deletion at Chr 22: 32,085,572–32,090,063. (8.90 MB ZIP) [file pone.0003104.s005.zip › SuppFig5/12_20859912_20859936_res.emphase.LD.PNG]

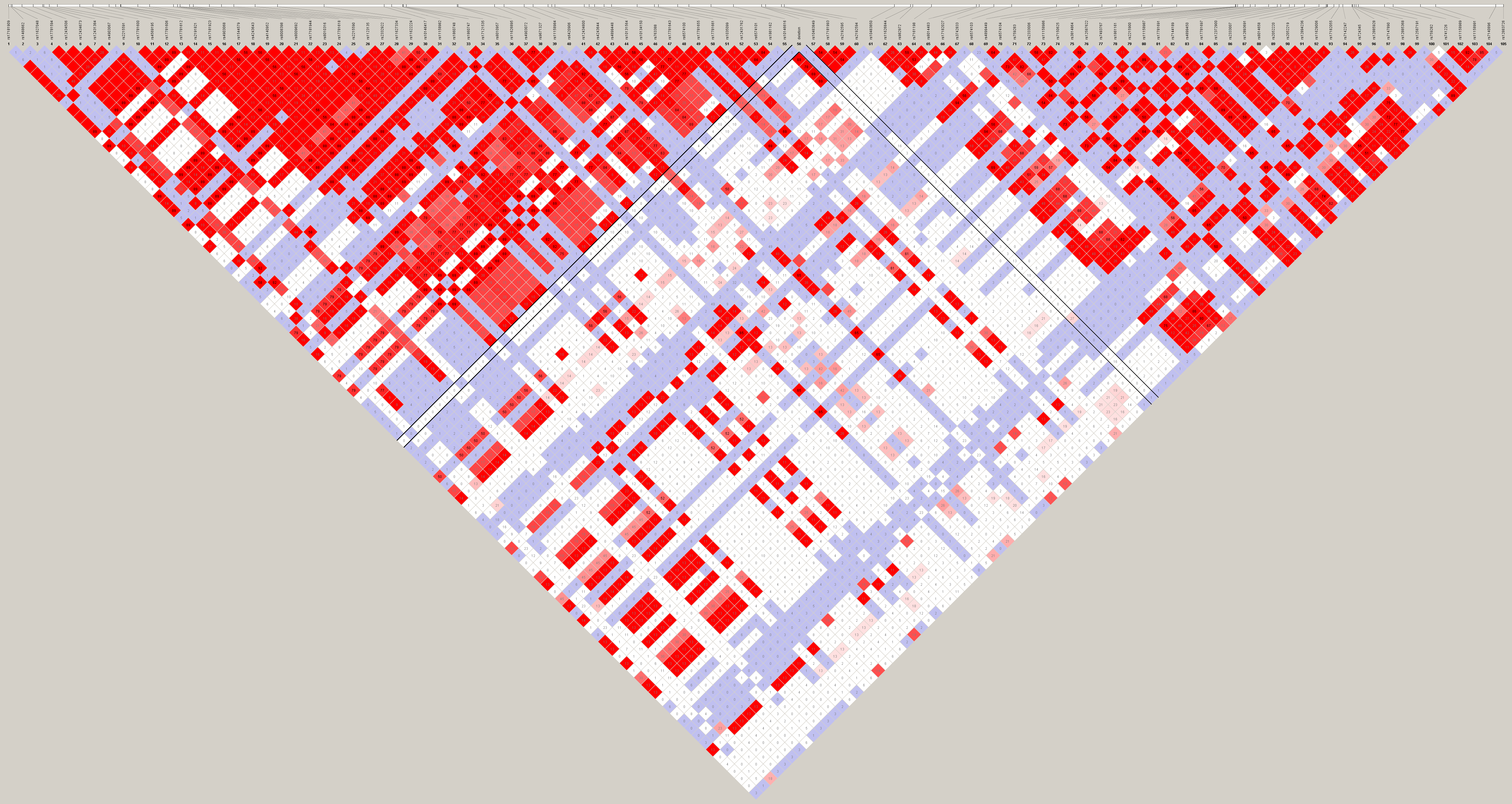

Supplement: Figure S5 — Plots show linkage disequilibrium (LD) of SNPs within 100 kb of each deletion. The default colouring scheme of Haploview is used, whereby positions are coloured white if LOD <2 and D' <1; blue if LOD <2 and D' = 1; shades of red as a function of D' if LOD≥2; bright red if D' = 1 and LOD ≥2. Numbers within the box refer to the r2 values between two given positions, and so are not directly connected to the colouring scheme. The solid black lines delineate the LD between the deletion and other markers in this region. a) Deletion at chr 1: 145,312,298–145,314,875; b) Deletion at Chr 2: 229,467,533–229,468,151; c) Deletion at Chr 3: 181,137,036–181,137,500; d) Deletion at Chr 4: 98,573,315–98,578,237; e) Deletion at Chr 5: 65,479,440–65,479,975; f) Deletion at Chr 5: 78,145,556–78,147,626; g) Deletion at Chr 6: 24,433,346–24,435,791; h) Deletion at Chr 6: 34,425,089–34,427,582; i) Deletion at Chr 6: 162,645,085–162,645,903; j) Deletion at Chr 7: 82,856,584–82,857,509; k) Deletion at Chr 12: 20,859,912–20,859,936; l) Deletion at Chr 14: 72,402,707–72,403,561; m) Deletion at Chr 14: 72,615,524–72,616,685; n) Deletion at Chr 15: 83,858,016–83,860,206; o) Deletion at Chr 16: 22,955,277–22,957,032; p) Deletion at Chr 16: 56,282,301–56,285,908; q) Deletion at Chr 16: 76,115,174–76,115,188; r) Deletion at Chr 16: 88,089,521–88,095,227; s) Deletion at Chr 19: 35,979,321–35,981,593; t) Deletion at Chr 22: 32,085,572–32,090,063. (8.90 MB ZIP) [file pone.0003104.s005.zip › SuppFig5/14_72402707_72403561_res.emphase.LD.PNG]

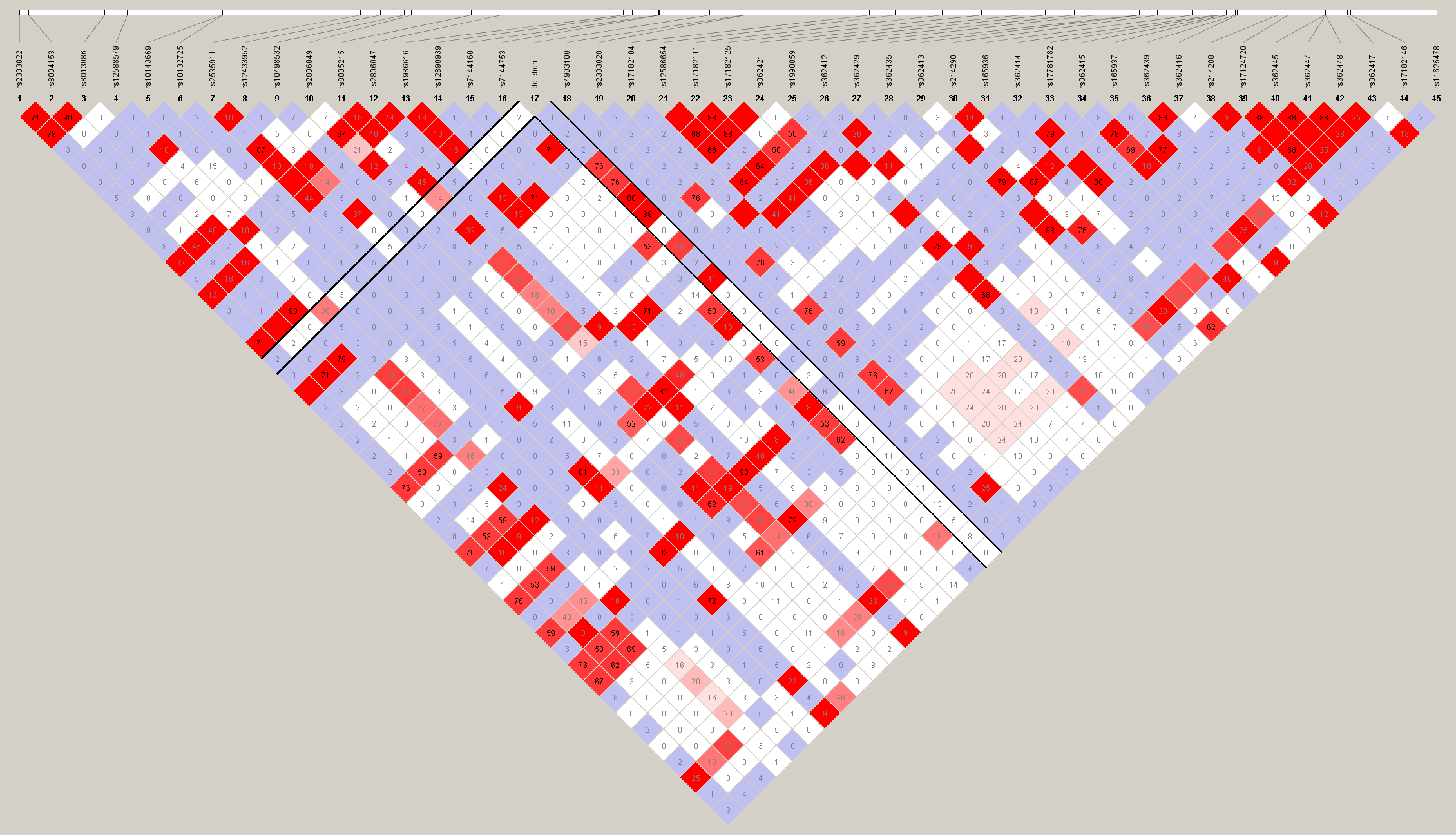

Supplement: Figure S5 — Plots show linkage disequilibrium (LD) of SNPs within 100 kb of each deletion. The default colouring scheme of Haploview is used, whereby positions are coloured white if LOD <2 and D' <1; blue if LOD <2 and D' = 1; shades of red as a function of D' if LOD≥2; bright red if D' = 1 and LOD ≥2. Numbers within the box refer to the r2 values between two given positions, and so are not directly connected to the colouring scheme. The solid black lines delineate the LD between the deletion and other markers in this region. a) Deletion at chr 1: 145,312,298–145,314,875; b) Deletion at Chr 2: 229,467,533–229,468,151; c) Deletion at Chr 3: 181,137,036–181,137,500; d) Deletion at Chr 4: 98,573,315–98,578,237; e) Deletion at Chr 5: 65,479,440–65,479,975; f) Deletion at Chr 5: 78,145,556–78,147,626; g) Deletion at Chr 6: 24,433,346–24,435,791; h) Deletion at Chr 6: 34,425,089–34,427,582; i) Deletion at Chr 6: 162,645,085–162,645,903; j) Deletion at Chr 7: 82,856,584–82,857,509; k) Deletion at Chr 12: 20,859,912–20,859,936; l) Deletion at Chr 14: 72,402,707–72,403,561; m) Deletion at Chr 14: 72,615,524–72,616,685; n) Deletion at Chr 15: 83,858,016–83,860,206; o) Deletion at Chr 16: 22,955,277–22,957,032; p) Deletion at Chr 16: 56,282,301–56,285,908; q) Deletion at Chr 16: 76,115,174–76,115,188; r) Deletion at Chr 16: 88,089,521–88,095,227; s) Deletion at Chr 19: 35,979,321–35,981,593; t) Deletion at Chr 22: 32,085,572–32,090,063. (8.90 MB ZIP) [file pone.0003104.s005.zip › SuppFig5/14_72615524_72616685_res.emphase.LD.PNG]

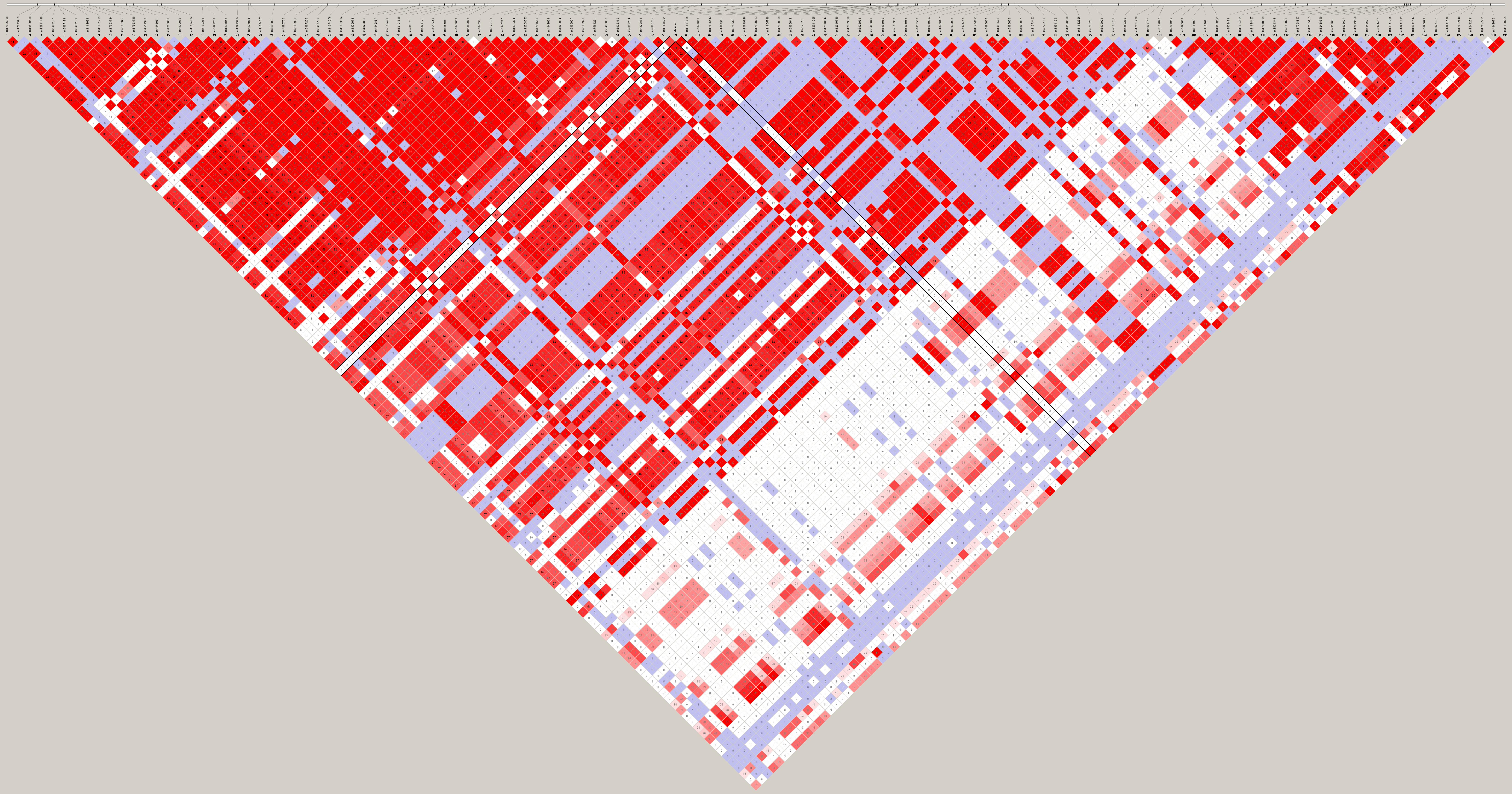

Supplement: Figure S5 — Plots show linkage disequilibrium (LD) of SNPs within 100 kb of each deletion. The default colouring scheme of Haploview is used, whereby positions are coloured white if LOD <2 and D' <1; blue if LOD <2 and D' = 1; shades of red as a function of D' if LOD≥2; bright red if D' = 1 and LOD ≥2. Numbers within the box refer to the r2 values between two given positions, and so are not directly connected to the colouring scheme. The solid black lines delineate the LD between the deletion and other markers in this region. a) Deletion at chr 1: 145,312,298–145,314,875; b) Deletion at Chr 2: 229,467,533–229,468,151; c) Deletion at Chr 3: 181,137,036–181,137,500; d) Deletion at Chr 4: 98,573,315–98,578,237; e) Deletion at Chr 5: 65,479,440–65,479,975; f) Deletion at Chr 5: 78,145,556–78,147,626; g) Deletion at Chr 6: 24,433,346–24,435,791; h) Deletion at Chr 6: 34,425,089–34,427,582; i) Deletion at Chr 6: 162,645,085–162,645,903; j) Deletion at Chr 7: 82,856,584–82,857,509; k) Deletion at Chr 12: 20,859,912–20,859,936; l) Deletion at Chr 14: 72,402,707–72,403,561; m) Deletion at Chr 14: 72,615,524–72,616,685; n) Deletion at Chr 15: 83,858,016–83,860,206; o) Deletion at Chr 16: 22,955,277–22,957,032; p) Deletion at Chr 16: 56,282,301–56,285,908; q) Deletion at Chr 16: 76,115,174–76,115,188; r) Deletion at Chr 16: 88,089,521–88,095,227; s) Deletion at Chr 19: 35,979,321–35,981,593; t) Deletion at Chr 22: 32,085,572–32,090,063. (8.90 MB ZIP) [file pone.0003104.s005.zip › SuppFig5/15_83858016_83860206_res.emphase.LD.PNG]

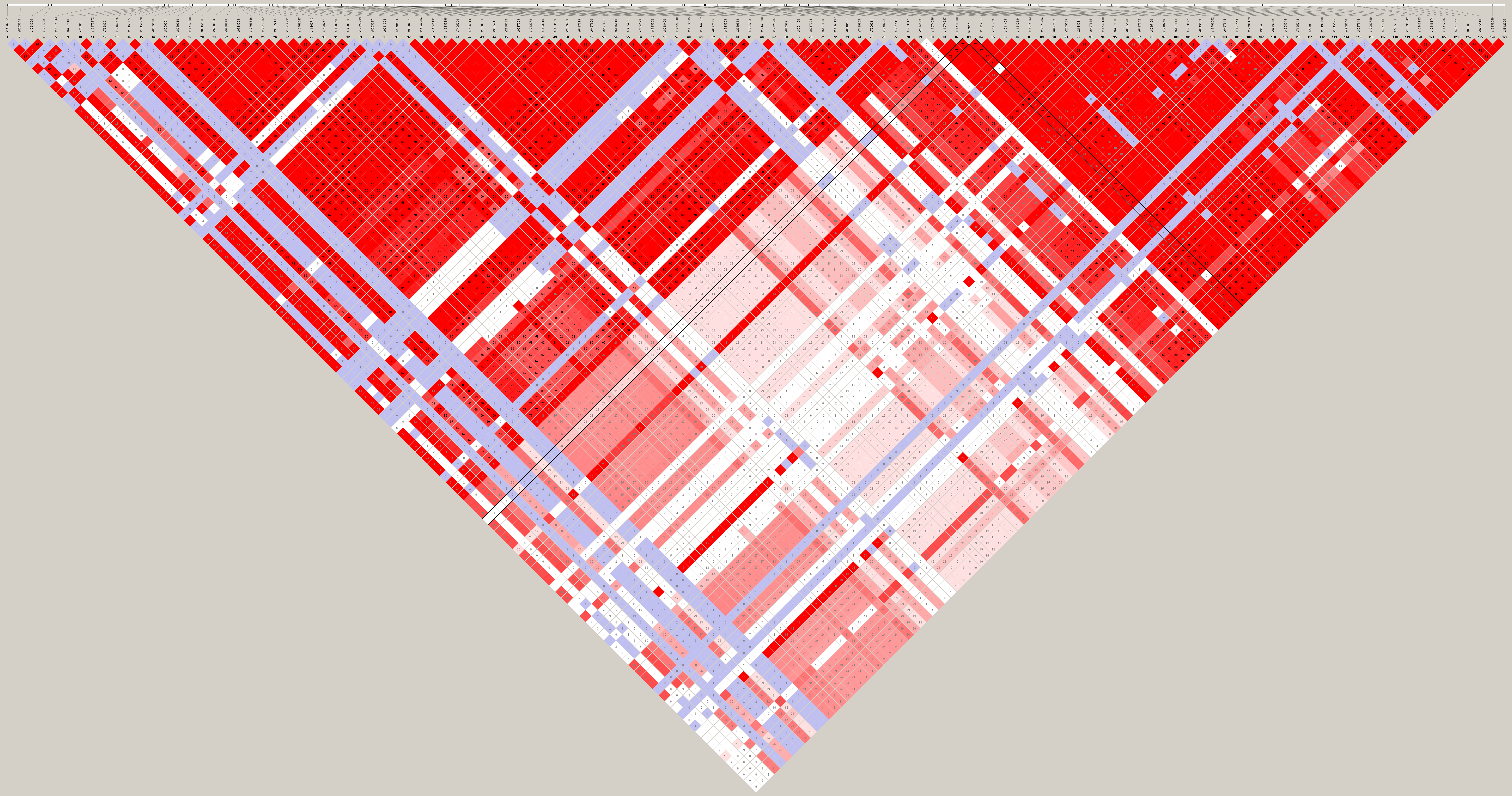

Supplement: Figure S5 — Plots show linkage disequilibrium (LD) of SNPs within 100 kb of each deletion. The default colouring scheme of Haploview is used, whereby positions are coloured white if LOD <2 and D' <1; blue if LOD <2 and D' = 1; shades of red as a function of D' if LOD≥2; bright red if D' = 1 and LOD ≥2. Numbers within the box refer to the r2 values between two given positions, and so are not directly connected to the colouring scheme. The solid black lines delineate the LD between the deletion and other markers in this region. a) Deletion at chr 1: 145,312,298–145,314,875; b) Deletion at Chr 2: 229,467,533–229,468,151; c) Deletion at Chr 3: 181,137,036–181,137,500; d) Deletion at Chr 4: 98,573,315–98,578,237; e) Deletion at Chr 5: 65,479,440–65,479,975; f) Deletion at Chr 5: 78,145,556–78,147,626; g) Deletion at Chr 6: 24,433,346–24,435,791; h) Deletion at Chr 6: 34,425,089–34,427,582; i) Deletion at Chr 6: 162,645,085–162,645,903; j) Deletion at Chr 7: 82,856,584–82,857,509; k) Deletion at Chr 12: 20,859,912–20,859,936; l) Deletion at Chr 14: 72,402,707–72,403,561; m) Deletion at Chr 14: 72,615,524–72,616,685; n) Deletion at Chr 15: 83,858,016–83,860,206; o) Deletion at Chr 16: 22,955,277–22,957,032; p) Deletion at Chr 16: 56,282,301–56,285,908; q) Deletion at Chr 16: 76,115,174–76,115,188; r) Deletion at Chr 16: 88,089,521–88,095,227; s) Deletion at Chr 19: 35,979,321–35,981,593; t) Deletion at Chr 22: 32,085,572–32,090,063. (8.90 MB ZIP) [file pone.0003104.s005.zip › SuppFig5/16_22955277_22957032_res.emphase.LD.PNG]

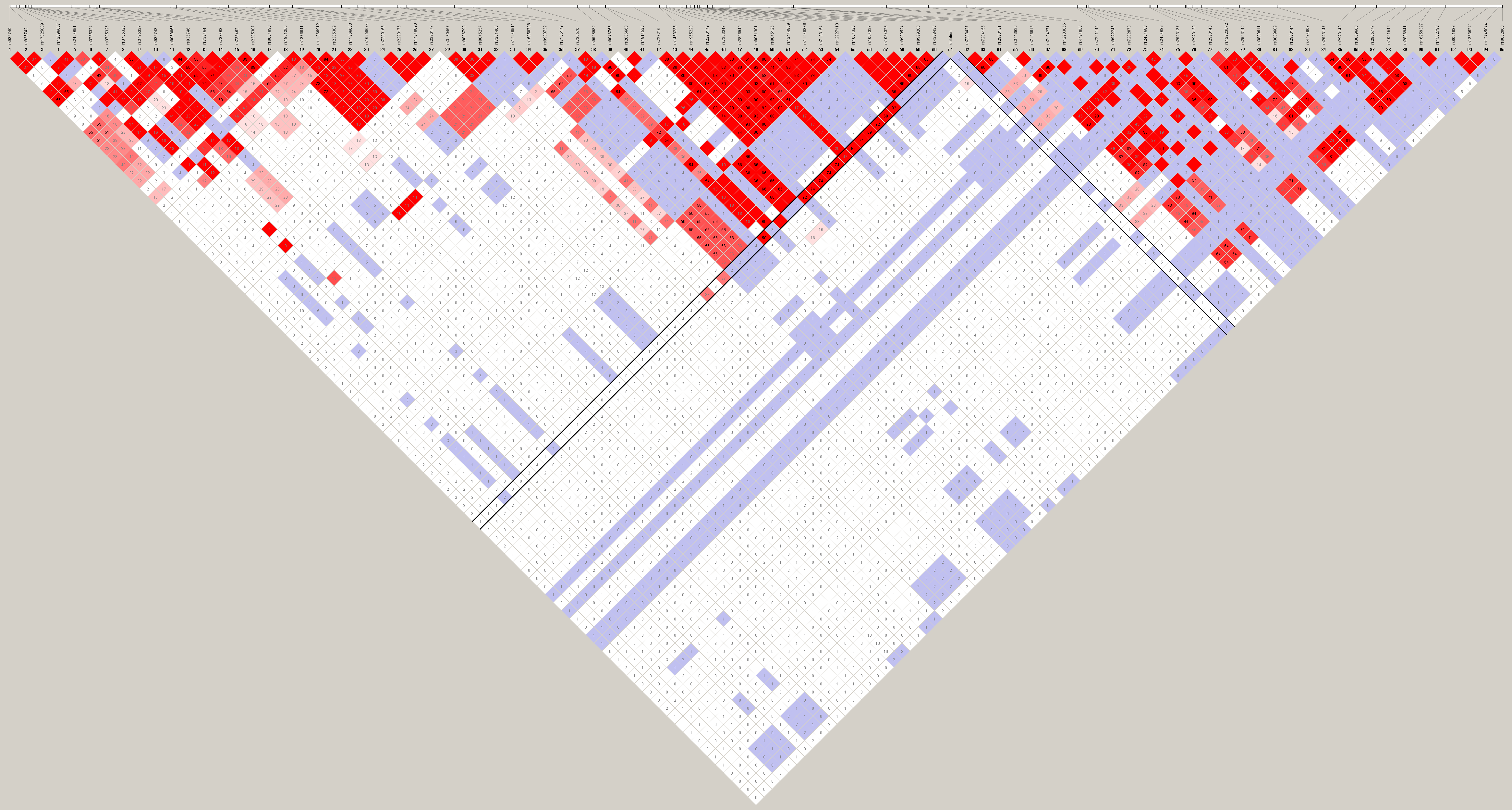

Supplement: Figure S5 — Plots show linkage disequilibrium (LD) of SNPs within 100 kb of each deletion. The default colouring scheme of Haploview is used, whereby positions are coloured white if LOD <2 and D' <1; blue if LOD <2 and D' = 1; shades of red as a function of D' if LOD≥2; bright red if D' = 1 and LOD ≥2. Numbers within the box refer to the r2 values between two given positions, and so are not directly connected to the colouring scheme. The solid black lines delineate the LD between the deletion and other markers in this region. a) Deletion at chr 1: 145,312,298–145,314,875; b) Deletion at Chr 2: 229,467,533–229,468,151; c) Deletion at Chr 3: 181,137,036–181,137,500; d) Deletion at Chr 4: 98,573,315–98,578,237; e) Deletion at Chr 5: 65,479,440–65,479,975; f) Deletion at Chr 5: 78,145,556–78,147,626; g) Deletion at Chr 6: 24,433,346–24,435,791; h) Deletion at Chr 6: 34,425,089–34,427,582; i) Deletion at Chr 6: 162,645,085–162,645,903; j) Deletion at Chr 7: 82,856,584–82,857,509; k) Deletion at Chr 12: 20,859,912–20,859,936; l) Deletion at Chr 14: 72,402,707–72,403,561; m) Deletion at Chr 14: 72,615,524–72,616,685; n) Deletion at Chr 15: 83,858,016–83,860,206; o) Deletion at Chr 16: 22,955,277–22,957,032; p) Deletion at Chr 16: 56,282,301–56,285,908; q) Deletion at Chr 16: 76,115,174–76,115,188; r) Deletion at Chr 16: 88,089,521–88,095,227; s) Deletion at Chr 19: 35,979,321–35,981,593; t) Deletion at Chr 22: 32,085,572–32,090,063. (8.90 MB ZIP) [file pone.0003104.s005.zip › SuppFig5/16_56282301_56285908_res.emphase.LD.PNG]

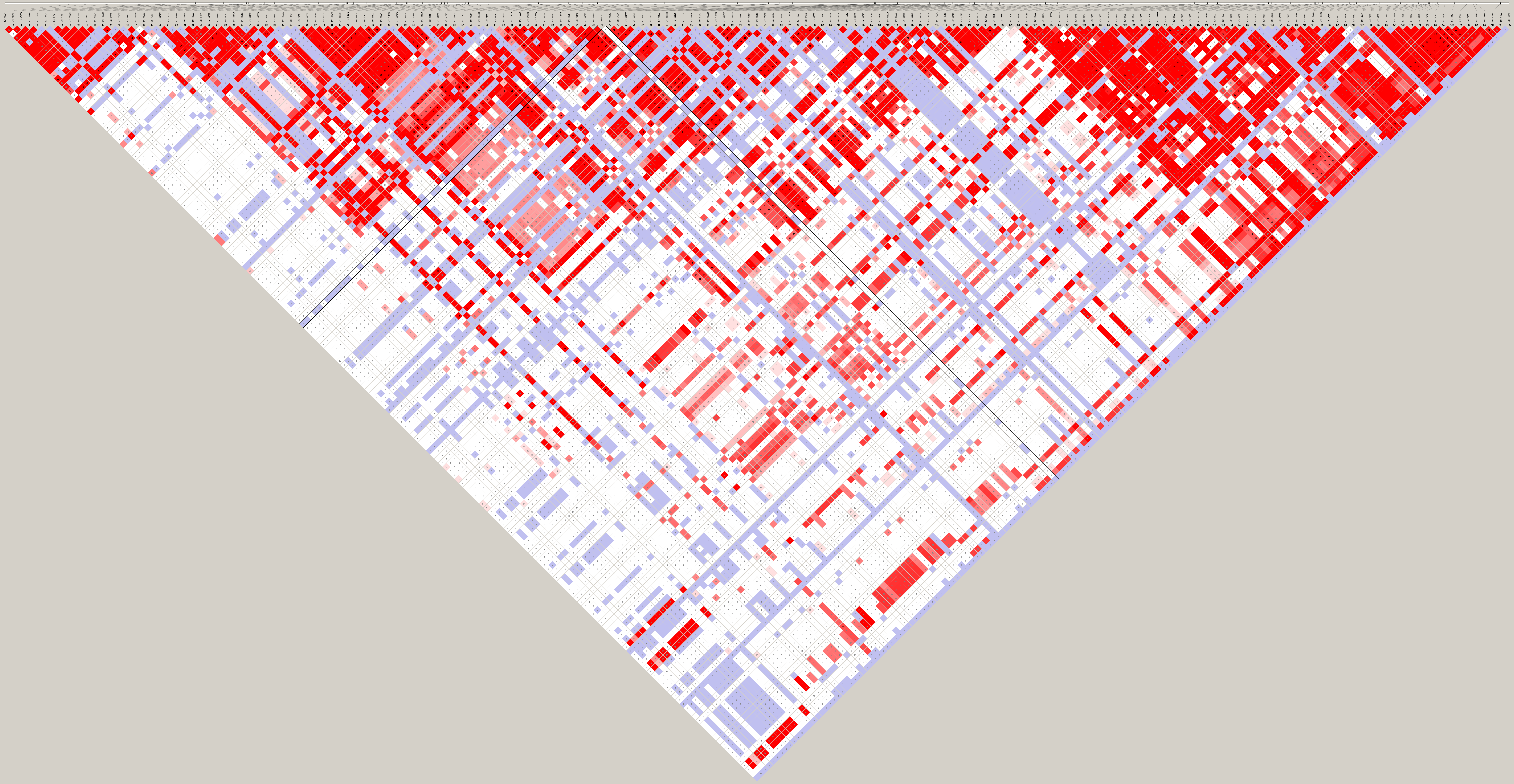

Supplement: Figure S5 — Plots show linkage disequilibrium (LD) of SNPs within 100 kb of each deletion. The default colouring scheme of Haploview is used, whereby positions are coloured white if LOD <2 and D' <1; blue if LOD <2 and D' = 1; shades of red as a function of D' if LOD≥2; bright red if D' = 1 and LOD ≥2. Numbers within the box refer to the r2 values between two given positions, and so are not directly connected to the colouring scheme. The solid black lines delineate the LD between the deletion and other markers in this region. a) Deletion at chr 1: 145,312,298–145,314,875; b) Deletion at Chr 2: 229,467,533–229,468,151; c) Deletion at Chr 3: 181,137,036–181,137,500; d) Deletion at Chr 4: 98,573,315–98,578,237; e) Deletion at Chr 5: 65,479,440–65,479,975; f) Deletion at Chr 5: 78,145,556–78,147,626; g) Deletion at Chr 6: 24,433,346–24,435,791; h) Deletion at Chr 6: 34,425,089–34,427,582; i) Deletion at Chr 6: 162,645,085–162,645,903; j) Deletion at Chr 7: 82,856,584–82,857,509; k) Deletion at Chr 12: 20,859,912–20,859,936; l) Deletion at Chr 14: 72,402,707–72,403,561; m) Deletion at Chr 14: 72,615,524–72,616,685; n) Deletion at Chr 15: 83,858,016–83,860,206; o) Deletion at Chr 16: 22,955,277–22,957,032; p) Deletion at Chr 16: 56,282,301–56,285,908; q) Deletion at Chr 16: 76,115,174–76,115,188; r) Deletion at Chr 16: 88,089,521–88,095,227; s) Deletion at Chr 19: 35,979,321–35,981,593; t) Deletion at Chr 22: 32,085,572–32,090,063. (8.90 MB ZIP) [file pone.0003104.s005.zip › SuppFig5/16_76115174_76115188_res.emphase.LD.PNG]

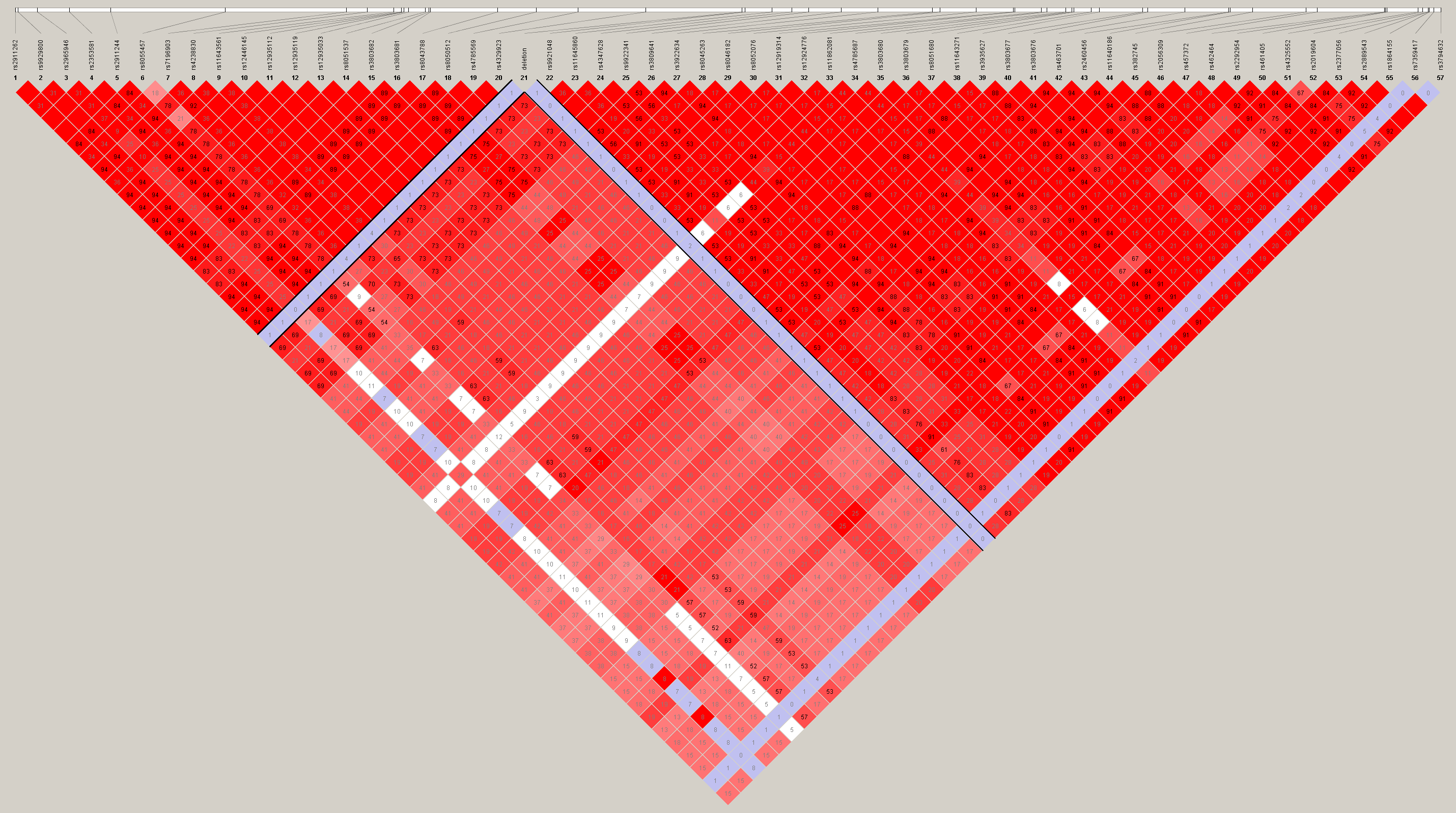

Supplement: Figure S5 — Plots show linkage disequilibrium (LD) of SNPs within 100 kb of each deletion. The default colouring scheme of Haploview is used, whereby positions are coloured white if LOD <2 and D' <1; blue if LOD <2 and D' = 1; shades of red as a function of D' if LOD≥2; bright red if D' = 1 and LOD ≥2. Numbers within the box refer to the r2 values between two given positions, and so are not directly connected to the colouring scheme. The solid black lines delineate the LD between the deletion and other markers in this region. a) Deletion at chr 1: 145,312,298–145,314,875; b) Deletion at Chr 2: 229,467,533–229,468,151; c) Deletion at Chr 3: 181,137,036–181,137,500; d) Deletion at Chr 4: 98,573,315–98,578,237; e) Deletion at Chr 5: 65,479,440–65,479,975; f) Deletion at Chr 5: 78,145,556–78,147,626; g) Deletion at Chr 6: 24,433,346–24,435,791; h) Deletion at Chr 6: 34,425,089–34,427,582; i) Deletion at Chr 6: 162,645,085–162,645,903; j) Deletion at Chr 7: 82,856,584–82,857,509; k) Deletion at Chr 12: 20,859,912–20,859,936; l) Deletion at Chr 14: 72,402,707–72,403,561; m) Deletion at Chr 14: 72,615,524–72,616,685; n) Deletion at Chr 15: 83,858,016–83,860,206; o) Deletion at Chr 16: 22,955,277–22,957,032; p) Deletion at Chr 16: 56,282,301–56,285,908; q) Deletion at Chr 16: 76,115,174–76,115,188; r) Deletion at Chr 16: 88,089,521–88,095,227; s) Deletion at Chr 19: 35,979,321–35,981,593; t) Deletion at Chr 22: 32,085,572–32,090,063. (8.90 MB ZIP) [file pone.0003104.s005.zip › SuppFig5/16_88089521_88095227_res.emphase.LD.PNG]

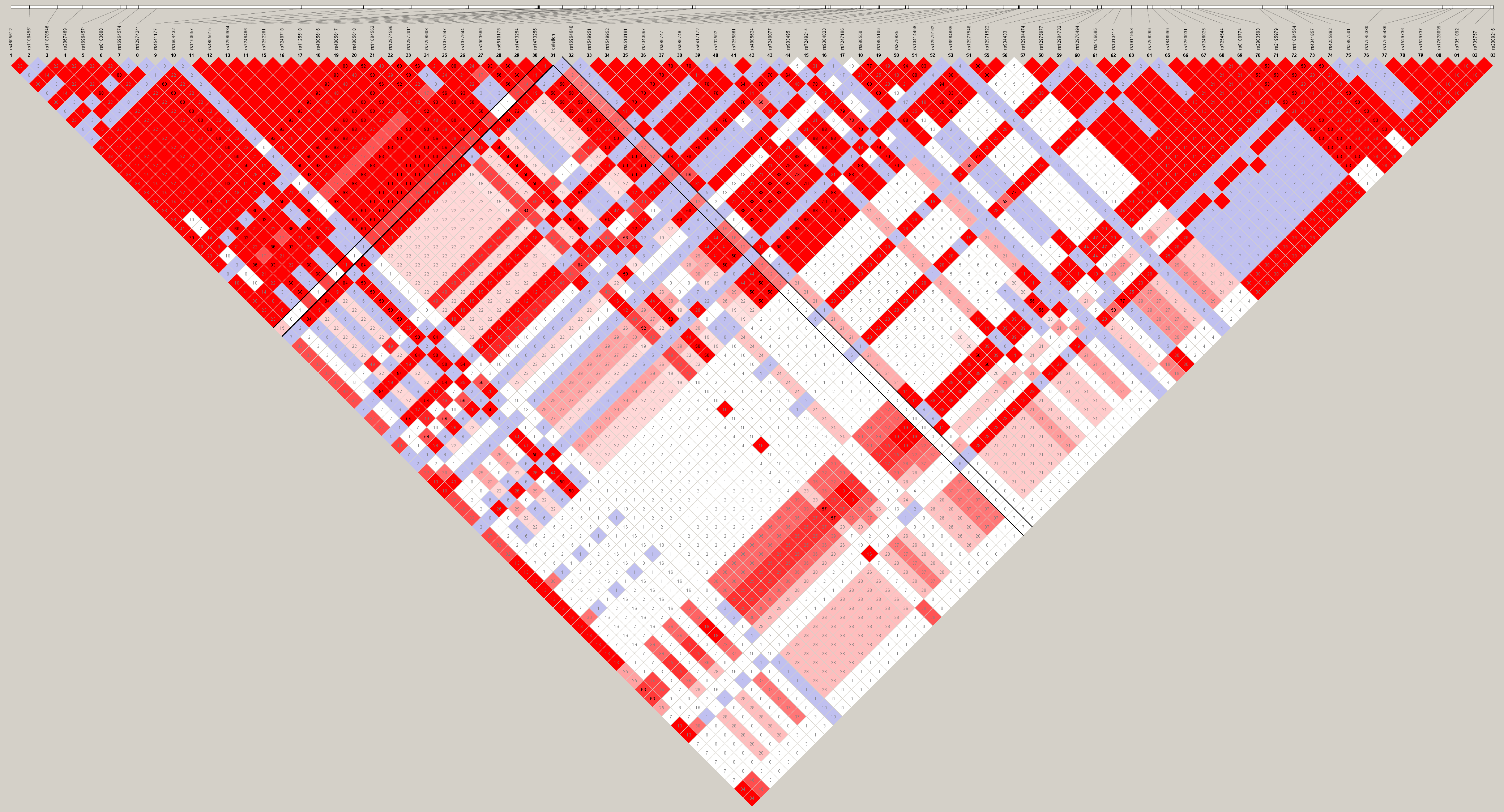

Supplement: Figure S5 — Plots show linkage disequilibrium (LD) of SNPs within 100 kb of each deletion. The default colouring scheme of Haploview is used, whereby positions are coloured white if LOD <2 and D' <1; blue if LOD <2 and D' = 1; shades of red as a function of D' if LOD≥2; bright red if D' = 1 and LOD ≥2. Numbers within the box refer to the r2 values between two given positions, and so are not directly connected to the colouring scheme. The solid black lines delineate the LD between the deletion and other markers in this region. a) Deletion at chr 1: 145,312,298–145,314,875; b) Deletion at Chr 2: 229,467,533–229,468,151; c) Deletion at Chr 3: 181,137,036–181,137,500; d) Deletion at Chr 4: 98,573,315–98,578,237; e) Deletion at Chr 5: 65,479,440–65,479,975; f) Deletion at Chr 5: 78,145,556–78,147,626; g) Deletion at Chr 6: 24,433,346–24,435,791; h) Deletion at Chr 6: 34,425,089–34,427,582; i) Deletion at Chr 6: 162,645,085–162,645,903; j) Deletion at Chr 7: 82,856,584–82,857,509; k) Deletion at Chr 12: 20,859,912–20,859,936; l) Deletion at Chr 14: 72,402,707–72,403,561; m) Deletion at Chr 14: 72,615,524–72,616,685; n) Deletion at Chr 15: 83,858,016–83,860,206; o) Deletion at Chr 16: 22,955,277–22,957,032; p) Deletion at Chr 16: 56,282,301–56,285,908; q) Deletion at Chr 16: 76,115,174–76,115,188; r) Deletion at Chr 16: 88,089,521–88,095,227; s) Deletion at Chr 19: 35,979,321–35,981,593; t) Deletion at Chr 22: 32,085,572–32,090,063. (8.90 MB ZIP) [file pone.0003104.s005.zip › SuppFig5/19_35979321_35981593_res.emphase.LD.PNG]

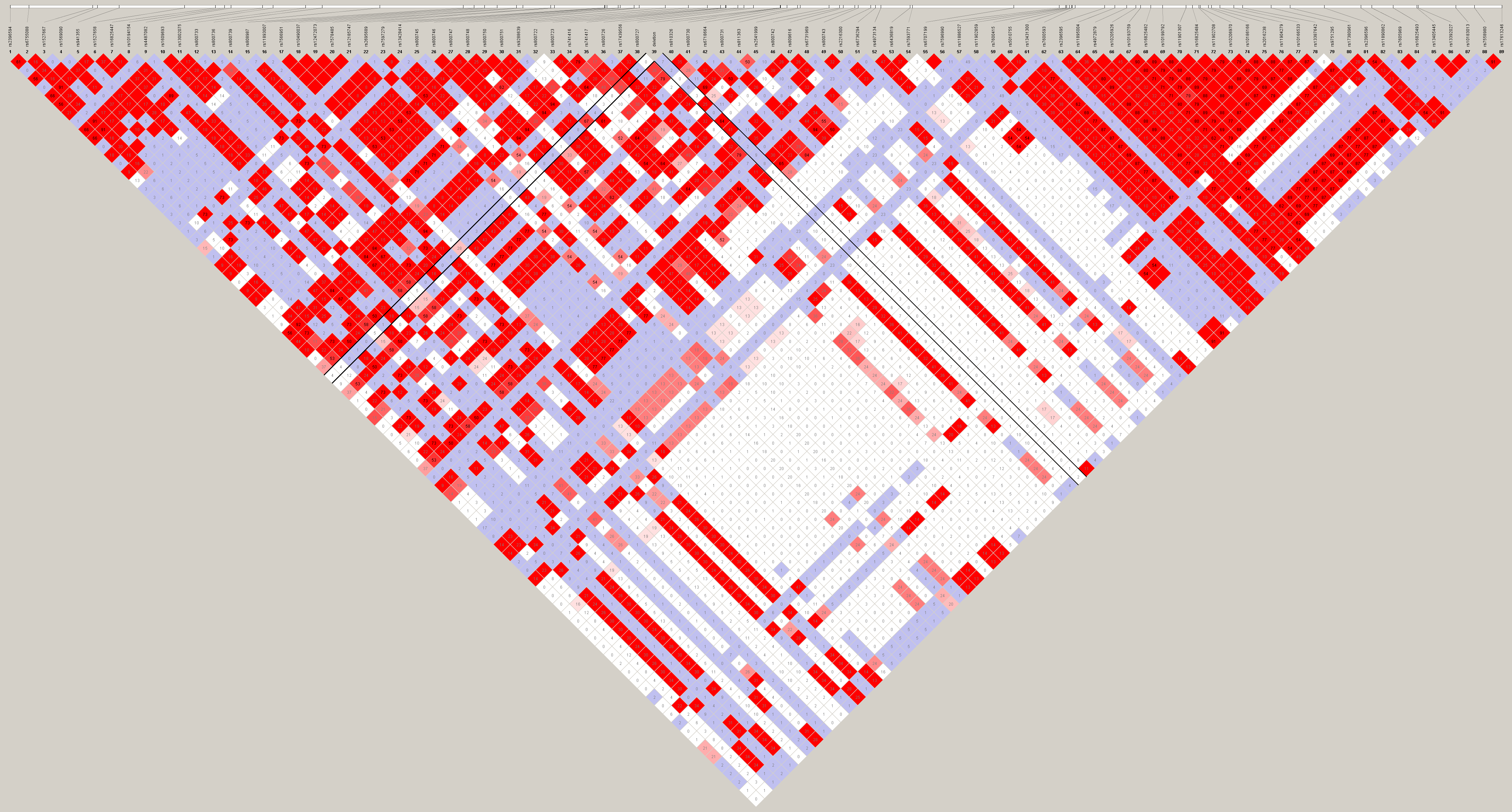

Supplement: Figure S5 — Plots show linkage disequilibrium (LD) of SNPs within 100 kb of each deletion. The default colouring scheme of Haploview is used, whereby positions are coloured white if LOD <2 and D' <1; blue if LOD <2 and D' = 1; shades of red as a function of D' if LOD≥2; bright red if D' = 1 and LOD ≥2. Numbers within the box refer to the r2 values between two given positions, and so are not directly connected to the colouring scheme. The solid black lines delineate the LD between the deletion and other markers in this region. a) Deletion at chr 1: 145,312,298–145,314,875; b) Deletion at Chr 2: 229,467,533–229,468,151; c) Deletion at Chr 3: 181,137,036–181,137,500; d) Deletion at Chr 4: 98,573,315–98,578,237; e) Deletion at Chr 5: 65,479,440–65,479,975; f) Deletion at Chr 5: 78,145,556–78,147,626; g) Deletion at Chr 6: 24,433,346–24,435,791; h) Deletion at Chr 6: 34,425,089–34,427,582; i) Deletion at Chr 6: 162,645,085–162,645,903; j) Deletion at Chr 7: 82,856,584–82,857,509; k) Deletion at Chr 12: 20,859,912–20,859,936; l) Deletion at Chr 14: 72,402,707–72,403,561; m) Deletion at Chr 14: 72,615,524–72,616,685; n) Deletion at Chr 15: 83,858,016–83,860,206; o) Deletion at Chr 16: 22,955,277–22,957,032; p) Deletion at Chr 16: 56,282,301–56,285,908; q) Deletion at Chr 16: 76,115,174–76,115,188; r) Deletion at Chr 16: 88,089,521–88,095,227; s) Deletion at Chr 19: 35,979,321–35,981,593; t) Deletion at Chr 22: 32,085,572–32,090,063. (8.90 MB ZIP) [file pone.0003104.s005.zip › SuppFig5/2_229467533_229468151_res.emphase.LD.PNG]

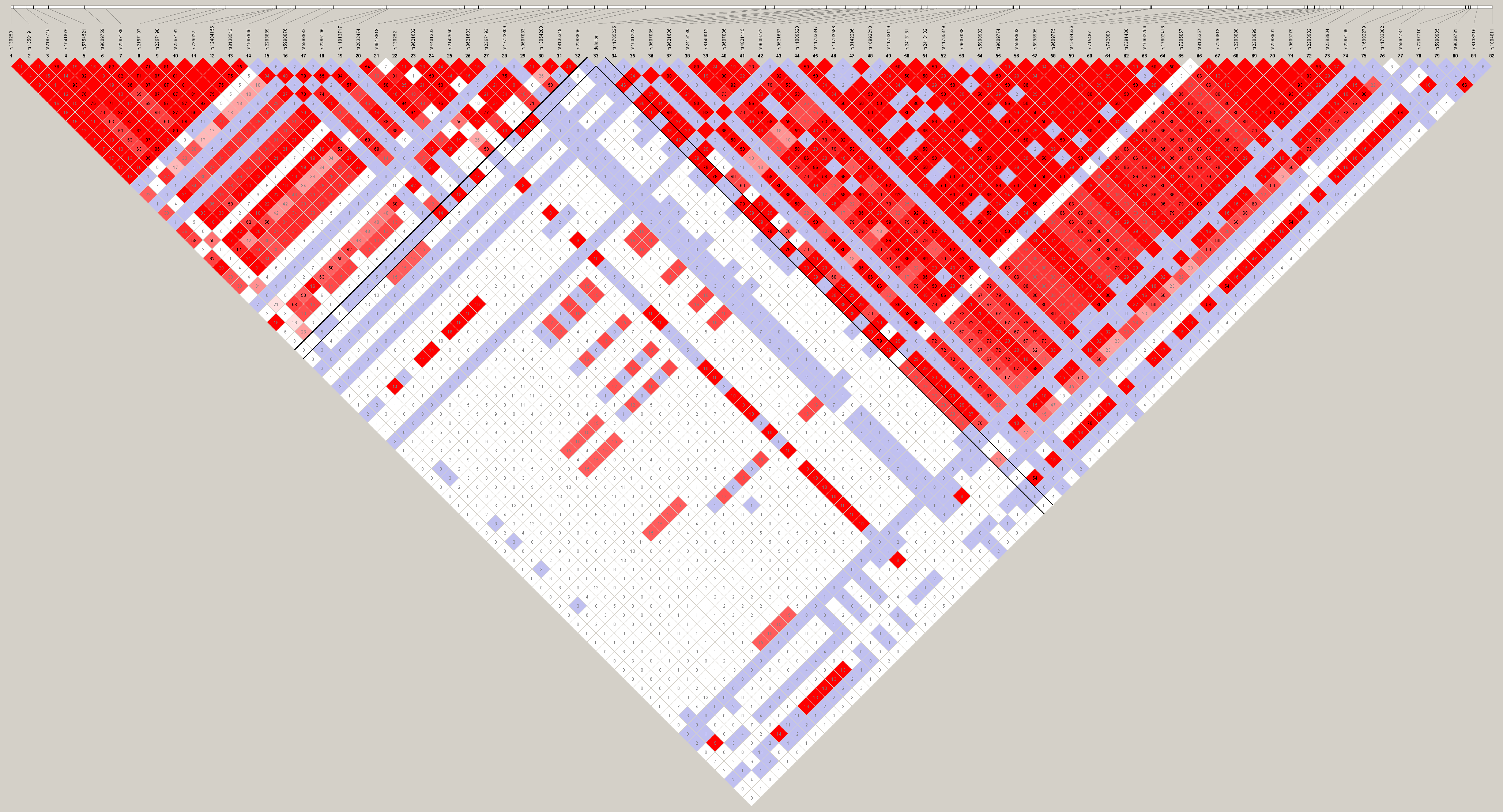

Supplement: Figure S5 — Plots show linkage disequilibrium (LD) of SNPs within 100 kb of each deletion. The default colouring scheme of Haploview is used, whereby positions are coloured white if LOD <2 and D' <1; blue if LOD <2 and D' = 1; shades of red as a function of D' if LOD≥2; bright red if D' = 1 and LOD ≥2. Numbers within the box refer to the r2 values between two given positions, and so are not directly connected to the colouring scheme. The solid black lines delineate the LD between the deletion and other markers in this region. a) Deletion at chr 1: 145,312,298–145,314,875; b) Deletion at Chr 2: 229,467,533–229,468,151; c) Deletion at Chr 3: 181,137,036–181,137,500; d) Deletion at Chr 4: 98,573,315–98,578,237; e) Deletion at Chr 5: 65,479,440–65,479,975; f) Deletion at Chr 5: 78,145,556–78,147,626; g) Deletion at Chr 6: 24,433,346–24,435,791; h) Deletion at Chr 6: 34,425,089–34,427,582; i) Deletion at Chr 6: 162,645,085–162,645,903; j) Deletion at Chr 7: 82,856,584–82,857,509; k) Deletion at Chr 12: 20,859,912–20,859,936; l) Deletion at Chr 14: 72,402,707–72,403,561; m) Deletion at Chr 14: 72,615,524–72,616,685; n) Deletion at Chr 15: 83,858,016–83,860,206; o) Deletion at Chr 16: 22,955,277–22,957,032; p) Deletion at Chr 16: 56,282,301–56,285,908; q) Deletion at Chr 16: 76,115,174–76,115,188; r) Deletion at Chr 16: 88,089,521–88,095,227; s) Deletion at Chr 19: 35,979,321–35,981,593; t) Deletion at Chr 22: 32,085,572–32,090,063. (8.90 MB ZIP) [file pone.0003104.s005.zip › SuppFig5/22_32085572_32090063_res.emphase.LD.PNG]

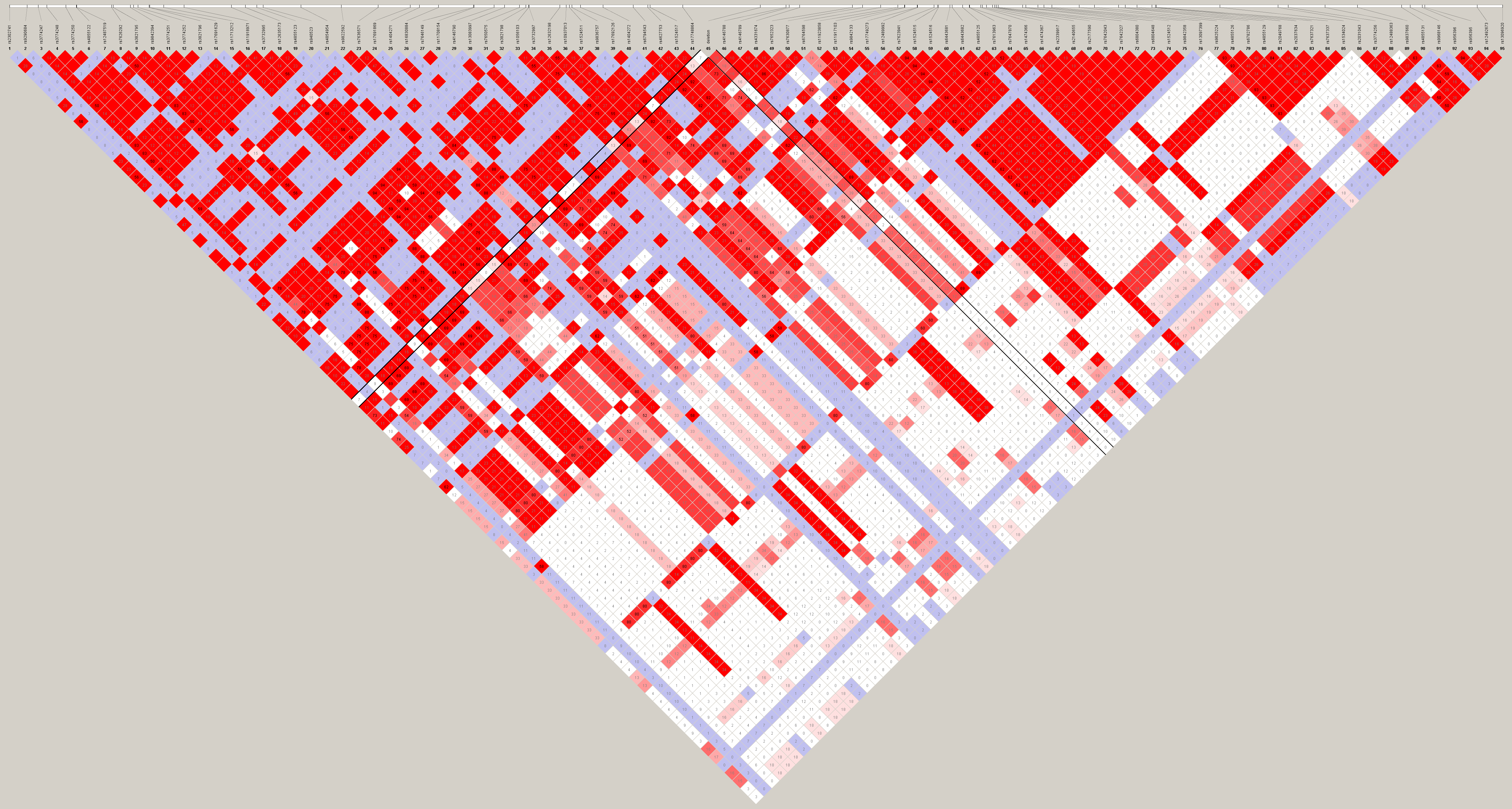

Supplement: Figure S5 — Plots show linkage disequilibrium (LD) of SNPs within 100 kb of each deletion. The default colouring scheme of Haploview is used, whereby positions are coloured white if LOD <2 and D' <1; blue if LOD <2 and D' = 1; shades of red as a function of D' if LOD≥2; bright red if D' = 1 and LOD ≥2. Numbers within the box refer to the r2 values between two given positions, and so are not directly connected to the colouring scheme. The solid black lines delineate the LD between the deletion and other markers in this region. a) Deletion at chr 1: 145,312,298–145,314,875; b) Deletion at Chr 2: 229,467,533–229,468,151; c) Deletion at Chr 3: 181,137,036–181,137,500; d) Deletion at Chr 4: 98,573,315–98,578,237; e) Deletion at Chr 5: 65,479,440–65,479,975; f) Deletion at Chr 5: 78,145,556–78,147,626; g) Deletion at Chr 6: 24,433,346–24,435,791; h) Deletion at Chr 6: 34,425,089–34,427,582; i) Deletion at Chr 6: 162,645,085–162,645,903; j) Deletion at Chr 7: 82,856,584–82,857,509; k) Deletion at Chr 12: 20,859,912–20,859,936; l) Deletion at Chr 14: 72,402,707–72,403,561; m) Deletion at Chr 14: 72,615,524–72,616,685; n) Deletion at Chr 15: 83,858,016–83,860,206; o) Deletion at Chr 16: 22,955,277–22,957,032; p) Deletion at Chr 16: 56,282,301–56,285,908; q) Deletion at Chr 16: 76,115,174–76,115,188; r) Deletion at Chr 16: 88,089,521–88,095,227; s) Deletion at Chr 19: 35,979,321–35,981,593; t) Deletion at Chr 22: 32,085,572–32,090,063. (8.90 MB ZIP) [file pone.0003104.s005.zip › SuppFig5/3_181137036_181137036_res.emphase.LD.PNG]

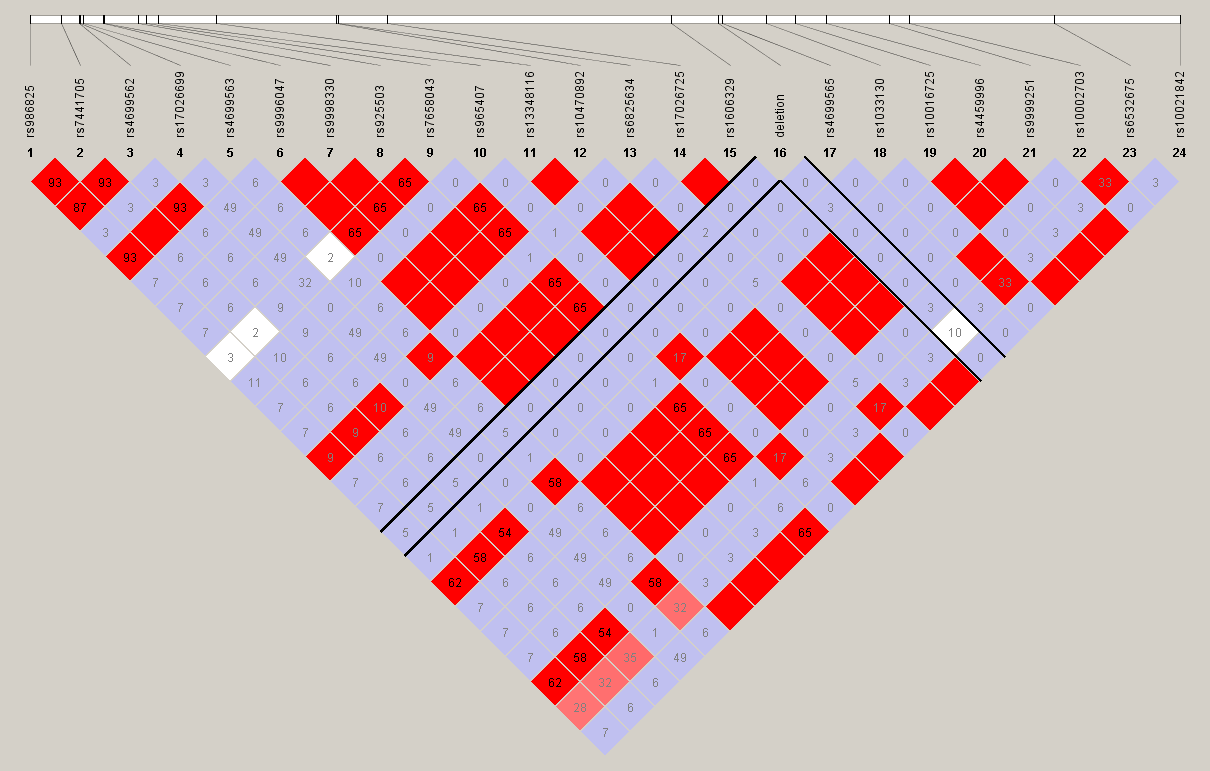

Supplement: Figure S5 — Plots show linkage disequilibrium (LD) of SNPs within 100 kb of each deletion. The default colouring scheme of Haploview is used, whereby positions are coloured white if LOD <2 and D' <1; blue if LOD <2 and D' = 1; shades of red as a function of D' if LOD≥2; bright red if D' = 1 and LOD ≥2. Numbers within the box refer to the r2 values between two given positions, and so are not directly connected to the colouring scheme. The solid black lines delineate the LD between the deletion and other markers in this region. a) Deletion at chr 1: 145,312,298–145,314,875; b) Deletion at Chr 2: 229,467,533–229,468,151; c) Deletion at Chr 3: 181,137,036–181,137,500; d) Deletion at Chr 4: 98,573,315–98,578,237; e) Deletion at Chr 5: 65,479,440–65,479,975; f) Deletion at Chr 5: 78,145,556–78,147,626; g) Deletion at Chr 6: 24,433,346–24,435,791; h) Deletion at Chr 6: 34,425,089–34,427,582; i) Deletion at Chr 6: 162,645,085–162,645,903; j) Deletion at Chr 7: 82,856,584–82,857,509; k) Deletion at Chr 12: 20,859,912–20,859,936; l) Deletion at Chr 14: 72,402,707–72,403,561; m) Deletion at Chr 14: 72,615,524–72,616,685; n) Deletion at Chr 15: 83,858,016–83,860,206; o) Deletion at Chr 16: 22,955,277–22,957,032; p) Deletion at Chr 16: 56,282,301–56,285,908; q) Deletion at Chr 16: 76,115,174–76,115,188; r) Deletion at Chr 16: 88,089,521–88,095,227; s) Deletion at Chr 19: 35,979,321–35,981,593; t) Deletion at Chr 22: 32,085,572–32,090,063. (8.90 MB ZIP) [file pone.0003104.s005.zip › SuppFig5/4_98573315_98578237_res.emphase.LD.PNG]

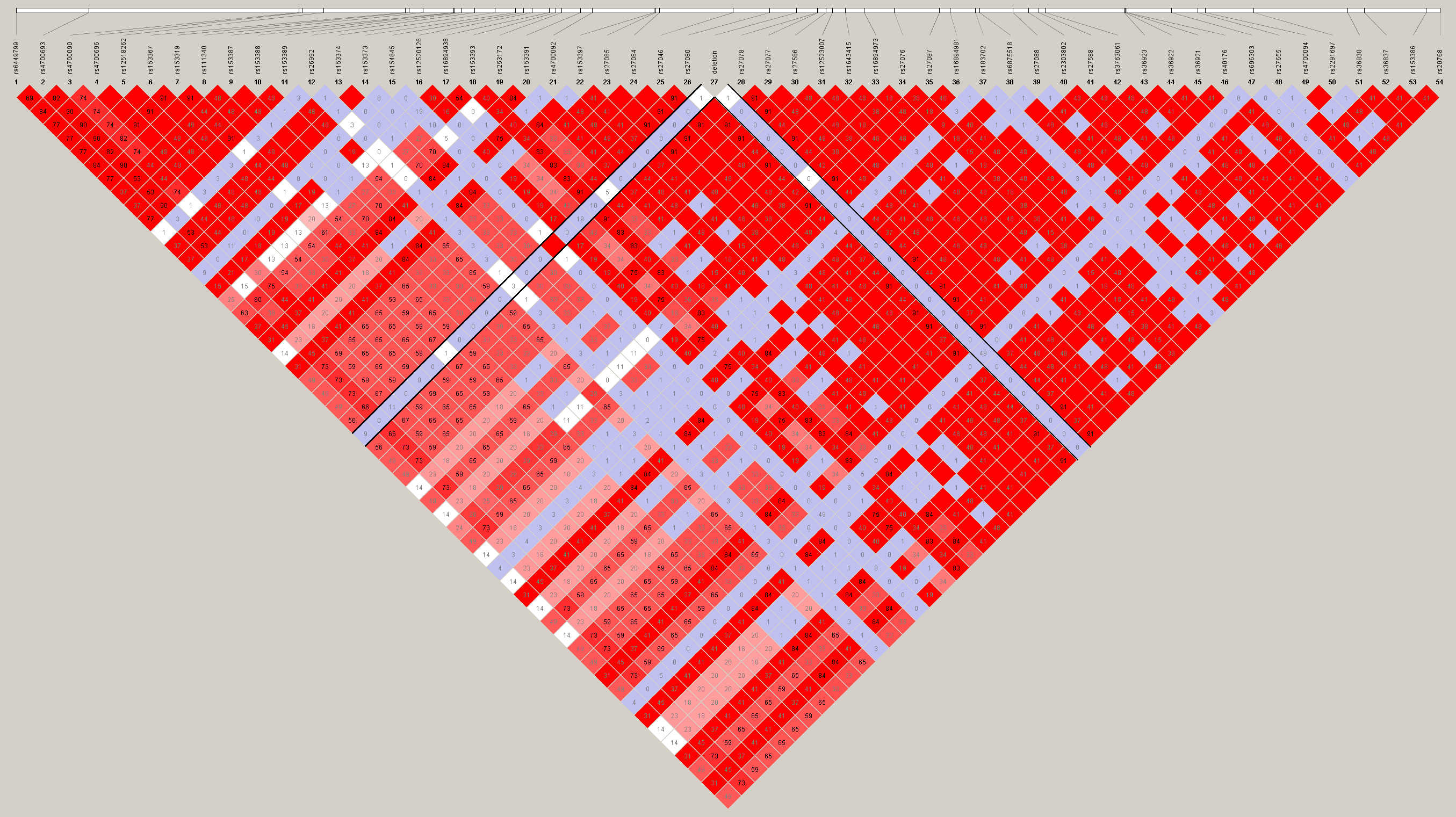

Supplement: Figure S5 — Plots show linkage disequilibrium (LD) of SNPs within 100 kb of each deletion. The default colouring scheme of Haploview is used, whereby positions are coloured white if LOD <2 and D' <1; blue if LOD <2 and D' = 1; shades of red as a function of D' if LOD≥2; bright red if D' = 1 and LOD ≥2. Numbers within the box refer to the r2 values between two given positions, and so are not directly connected to the colouring scheme. The solid black lines delineate the LD between the deletion and other markers in this region. a) Deletion at chr 1: 145,312,298–145,314,875; b) Deletion at Chr 2: 229,467,533–229,468,151; c) Deletion at Chr 3: 181,137,036–181,137,500; d) Deletion at Chr 4: 98,573,315–98,578,237; e) Deletion at Chr 5: 65,479,440–65,479,975; f) Deletion at Chr 5: 78,145,556–78,147,626; g) Deletion at Chr 6: 24,433,346–24,435,791; h) Deletion at Chr 6: 34,425,089–34,427,582; i) Deletion at Chr 6: 162,645,085–162,645,903; j) Deletion at Chr 7: 82,856,584–82,857,509; k) Deletion at Chr 12: 20,859,912–20,859,936; l) Deletion at Chr 14: 72,402,707–72,403,561; m) Deletion at Chr 14: 72,615,524–72,616,685; n) Deletion at Chr 15: 83,858,016–83,860,206; o) Deletion at Chr 16: 22,955,277–22,957,032; p) Deletion at Chr 16: 56,282,301–56,285,908; q) Deletion at Chr 16: 76,115,174–76,115,188; r) Deletion at Chr 16: 88,089,521–88,095,227; s) Deletion at Chr 19: 35,979,321–35,981,593; t) Deletion at Chr 22: 32,085,572–32,090,063. (8.90 MB ZIP) [file pone.0003104.s005.zip › SuppFig5/5_65479440_65479975_res.emphase.LD.PNG]

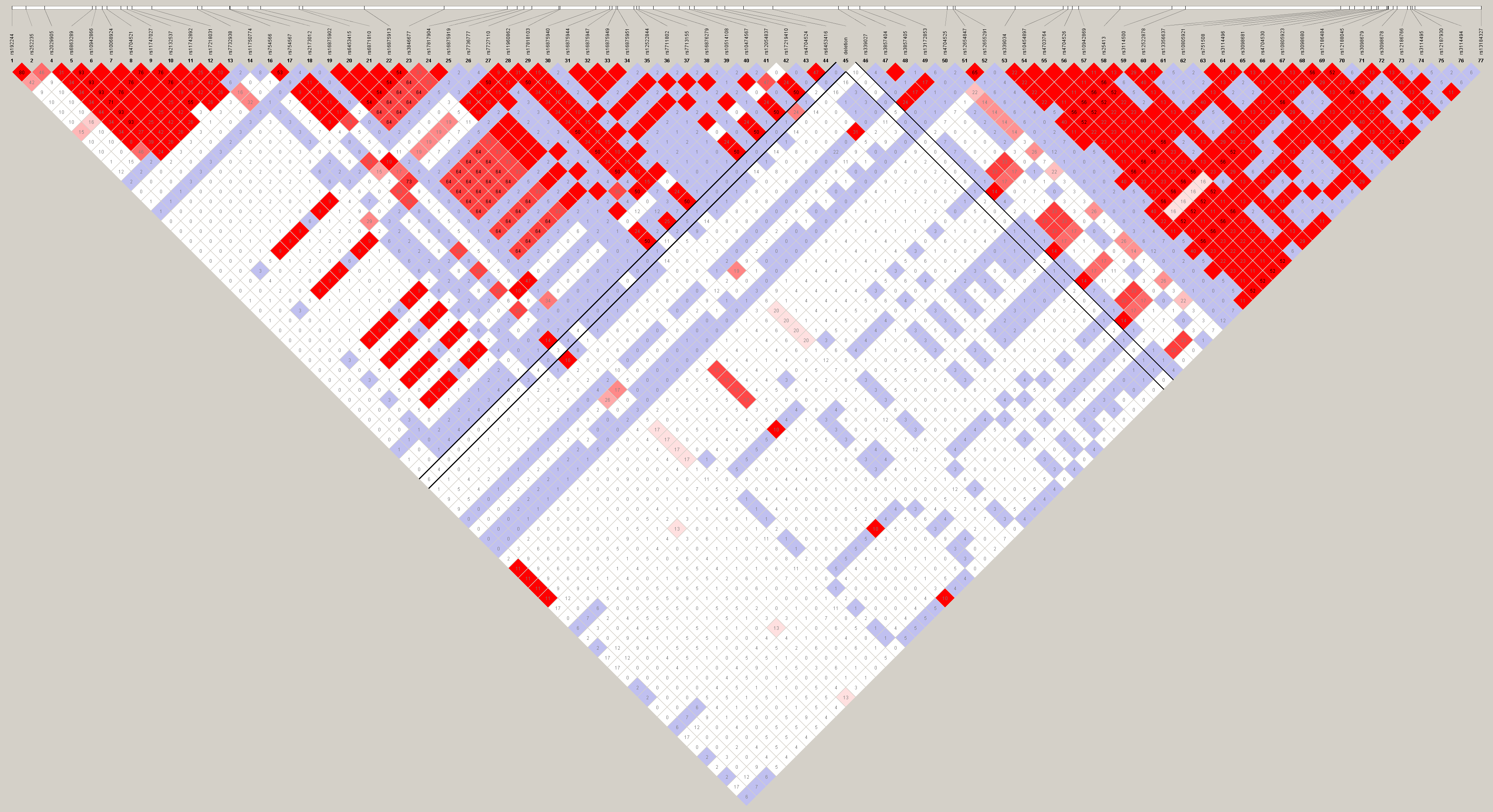

Supplement: Figure S5 — Plots show linkage disequilibrium (LD) of SNPs within 100 kb of each deletion. The default colouring scheme of Haploview is used, whereby positions are coloured white if LOD <2 and D' <1; blue if LOD <2 and D' = 1; shades of red as a function of D' if LOD≥2; bright red if D' = 1 and LOD ≥2. Numbers within the box refer to the r2 values between two given positions, and so are not directly connected to the colouring scheme. The solid black lines delineate the LD between the deletion and other markers in this region. a) Deletion at chr 1: 145,312,298–145,314,875; b) Deletion at Chr 2: 229,467,533–229,468,151; c) Deletion at Chr 3: 181,137,036–181,137,500; d) Deletion at Chr 4: 98,573,315–98,578,237; e) Deletion at Chr 5: 65,479,440–65,479,975; f) Deletion at Chr 5: 78,145,556–78,147,626; g) Deletion at Chr 6: 24,433,346–24,435,791; h) Deletion at Chr 6: 34,425,089–34,427,582; i) Deletion at Chr 6: 162,645,085–162,645,903; j) Deletion at Chr 7: 82,856,584–82,857,509; k) Deletion at Chr 12: 20,859,912–20,859,936; l) Deletion at Chr 14: 72,402,707–72,403,561; m) Deletion at Chr 14: 72,615,524–72,616,685; n) Deletion at Chr 15: 83,858,016–83,860,206; o) Deletion at Chr 16: 22,955,277–22,957,032; p) Deletion at Chr 16: 56,282,301–56,285,908; q) Deletion at Chr 16: 76,115,174–76,115,188; r) Deletion at Chr 16: 88,089,521–88,095,227; s) Deletion at Chr 19: 35,979,321–35,981,593; t) Deletion at Chr 22: 32,085,572–32,090,063. (8.90 MB ZIP) [file pone.0003104.s005.zip › SuppFig5/5_78145556_78147626_res.emphase.LD.PNG]

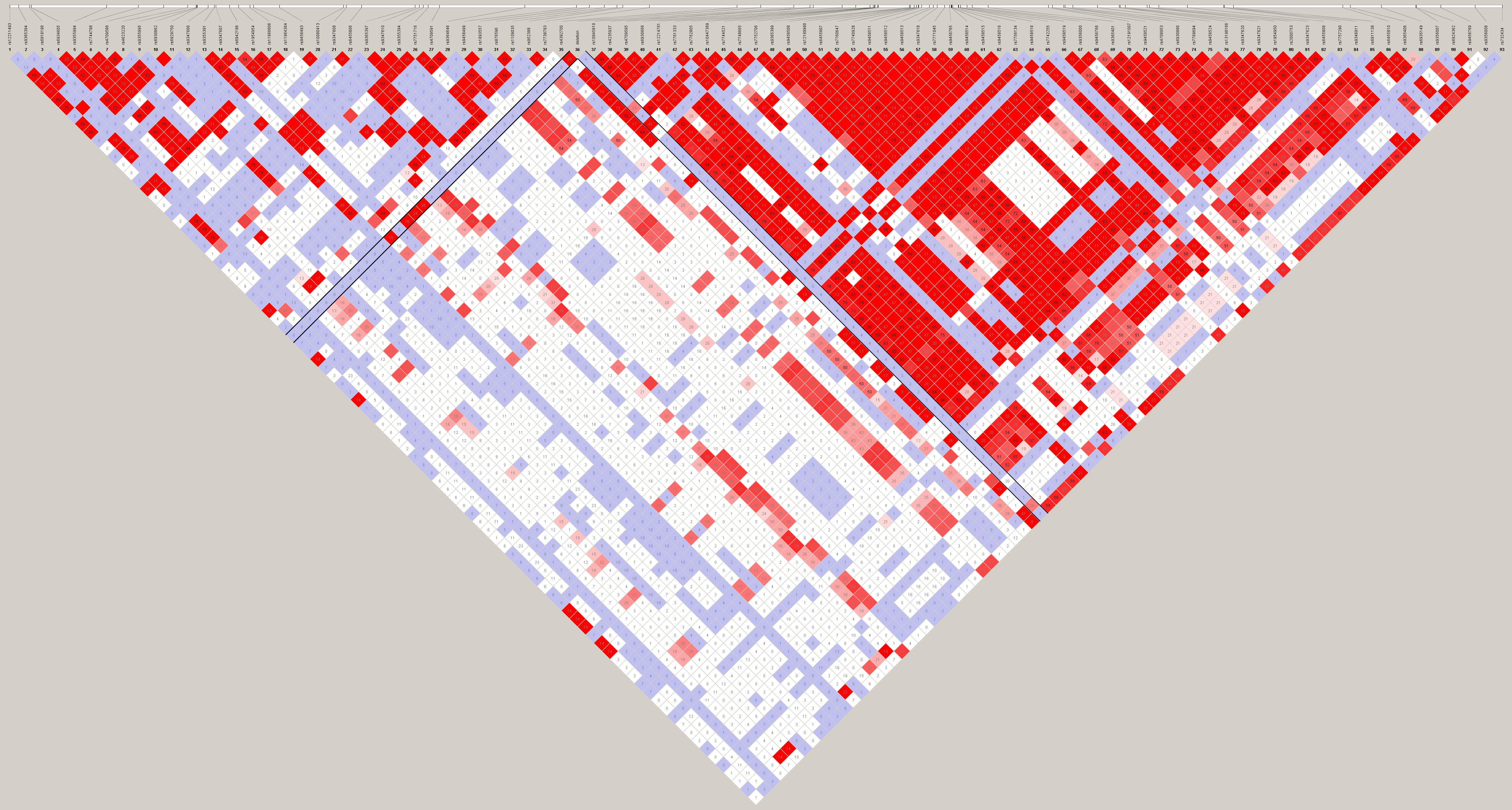

Supplement: Figure S5 — Plots show linkage disequilibrium (LD) of SNPs within 100 kb of each deletion. The default colouring scheme of Haploview is used, whereby positions are coloured white if LOD <2 and D' <1; blue if LOD <2 and D' = 1; shades of red as a function of D' if LOD≥2; bright red if D' = 1 and LOD ≥2. Numbers within the box refer to the r2 values between two given positions, and so are not directly connected to the colouring scheme. The solid black lines delineate the LD between the deletion and other markers in this region. a) Deletion at chr 1: 145,312,298–145,314,875; b) Deletion at Chr 2: 229,467,533–229,468,151; c) Deletion at Chr 3: 181,137,036–181,137,500; d) Deletion at Chr 4: 98,573,315–98,578,237; e) Deletion at Chr 5: 65,479,440–65,479,975; f) Deletion at Chr 5: 78,145,556–78,147,626; g) Deletion at Chr 6: 24,433,346–24,435,791; h) Deletion at Chr 6: 34,425,089–34,427,582; i) Deletion at Chr 6: 162,645,085–162,645,903; j) Deletion at Chr 7: 82,856,584–82,857,509; k) Deletion at Chr 12: 20,859,912–20,859,936; l) Deletion at Chr 14: 72,402,707–72,403,561; m) Deletion at Chr 14: 72,615,524–72,616,685; n) Deletion at Chr 15: 83,858,016–83,860,206; o) Deletion at Chr 16: 22,955,277–22,957,032; p) Deletion at Chr 16: 56,282,301–56,285,908; q) Deletion at Chr 16: 76,115,174–76,115,188; r) Deletion at Chr 16: 88,089,521–88,095,227; s) Deletion at Chr 19: 35,979,321–35,981,593; t) Deletion at Chr 22: 32,085,572–32,090,063. (8.90 MB ZIP) [file pone.0003104.s005.zip › SuppFig5/6_162645085_162645903_res.emphase.LD.PNG]

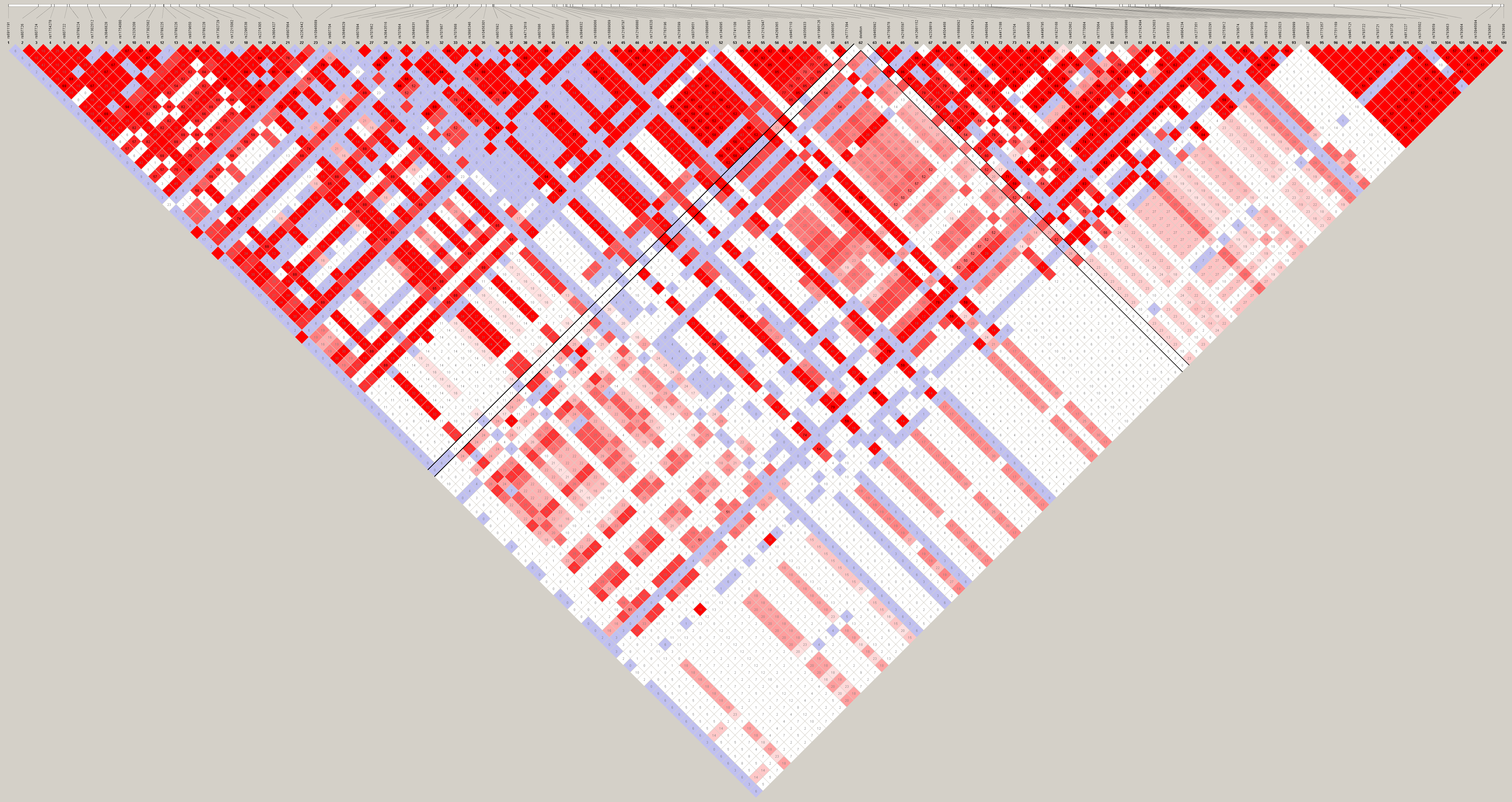

Supplement: Figure S5 — Plots show linkage disequilibrium (LD) of SNPs within 100 kb of each deletion. The default colouring scheme of Haploview is used, whereby positions are coloured white if LOD <2 and D' <1; blue if LOD <2 and D' = 1; shades of red as a function of D' if LOD≥2; bright red if D' = 1 and LOD ≥2. Numbers within the box refer to the r2 values between two given positions, and so are not directly connected to the colouring scheme. The solid black lines delineate the LD between the deletion and other markers in this region. a) Deletion at chr 1: 145,312,298–145,314,875; b) Deletion at Chr 2: 229,467,533–229,468,151; c) Deletion at Chr 3: 181,137,036–181,137,500; d) Deletion at Chr 4: 98,573,315–98,578,237; e) Deletion at Chr 5: 65,479,440–65,479,975; f) Deletion at Chr 5: 78,145,556–78,147,626; g) Deletion at Chr 6: 24,433,346–24,435,791; h) Deletion at Chr 6: 34,425,089–34,427,582; i) Deletion at Chr 6: 162,645,085–162,645,903; j) Deletion at Chr 7: 82,856,584–82,857,509; k) Deletion at Chr 12: 20,859,912–20,859,936; l) Deletion at Chr 14: 72,402,707–72,403,561; m) Deletion at Chr 14: 72,615,524–72,616,685; n) Deletion at Chr 15: 83,858,016–83,860,206; o) Deletion at Chr 16: 22,955,277–22,957,032; p) Deletion at Chr 16: 56,282,301–56,285,908; q) Deletion at Chr 16: 76,115,174–76,115,188; r) Deletion at Chr 16: 88,089,521–88,095,227; s) Deletion at Chr 19: 35,979,321–35,981,593; t) Deletion at Chr 22: 32,085,572–32,090,063. (8.90 MB ZIP) [file pone.0003104.s005.zip › SuppFig5/6_24433346_24435791_res.emphase.LD.PNG]

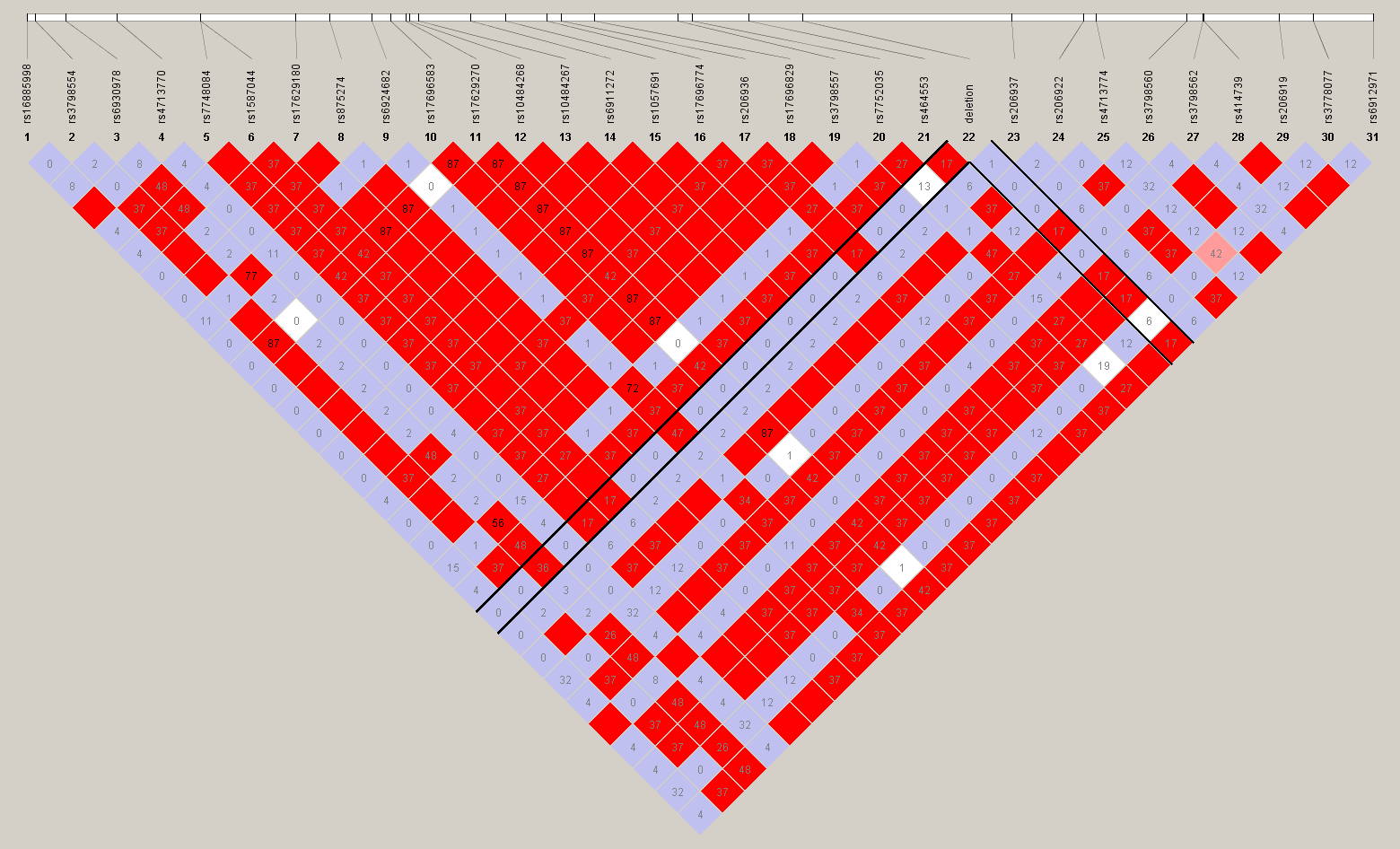

Supplement: Figure S5 — Plots show linkage disequilibrium (LD) of SNPs within 100 kb of each deletion. The default colouring scheme of Haploview is used, whereby positions are coloured white if LOD <2 and D' <1; blue if LOD <2 and D' = 1; shades of red as a function of D' if LOD≥2; bright red if D' = 1 and LOD ≥2. Numbers within the box refer to the r2 values between two given positions, and so are not directly connected to the colouring scheme. The solid black lines delineate the LD between the deletion and other markers in this region. a) Deletion at chr 1: 145,312,298–145,314,875; b) Deletion at Chr 2: 229,467,533–229,468,151; c) Deletion at Chr 3: 181,137,036–181,137,500; d) Deletion at Chr 4: 98,573,315–98,578,237; e) Deletion at Chr 5: 65,479,440–65,479,975; f) Deletion at Chr 5: 78,145,556–78,147,626; g) Deletion at Chr 6: 24,433,346–24,435,791; h) Deletion at Chr 6: 34,425,089–34,427,582; i) Deletion at Chr 6: 162,645,085–162,645,903; j) Deletion at Chr 7: 82,856,584–82,857,509; k) Deletion at Chr 12: 20,859,912–20,859,936; l) Deletion at Chr 14: 72,402,707–72,403,561; m) Deletion at Chr 14: 72,615,524–72,616,685; n) Deletion at Chr 15: 83,858,016–83,860,206; o) Deletion at Chr 16: 22,955,277–22,957,032; p) Deletion at Chr 16: 56,282,301–56,285,908; q) Deletion at Chr 16: 76,115,174–76,115,188; r) Deletion at Chr 16: 88,089,521–88,095,227; s) Deletion at Chr 19: 35,979,321–35,981,593; t) Deletion at Chr 22: 32,085,572–32,090,063. (8.90 MB ZIP) [file pone.0003104.s005.zip › SuppFig5/6_34425089_34427582_res.emphase.LD.PNG]

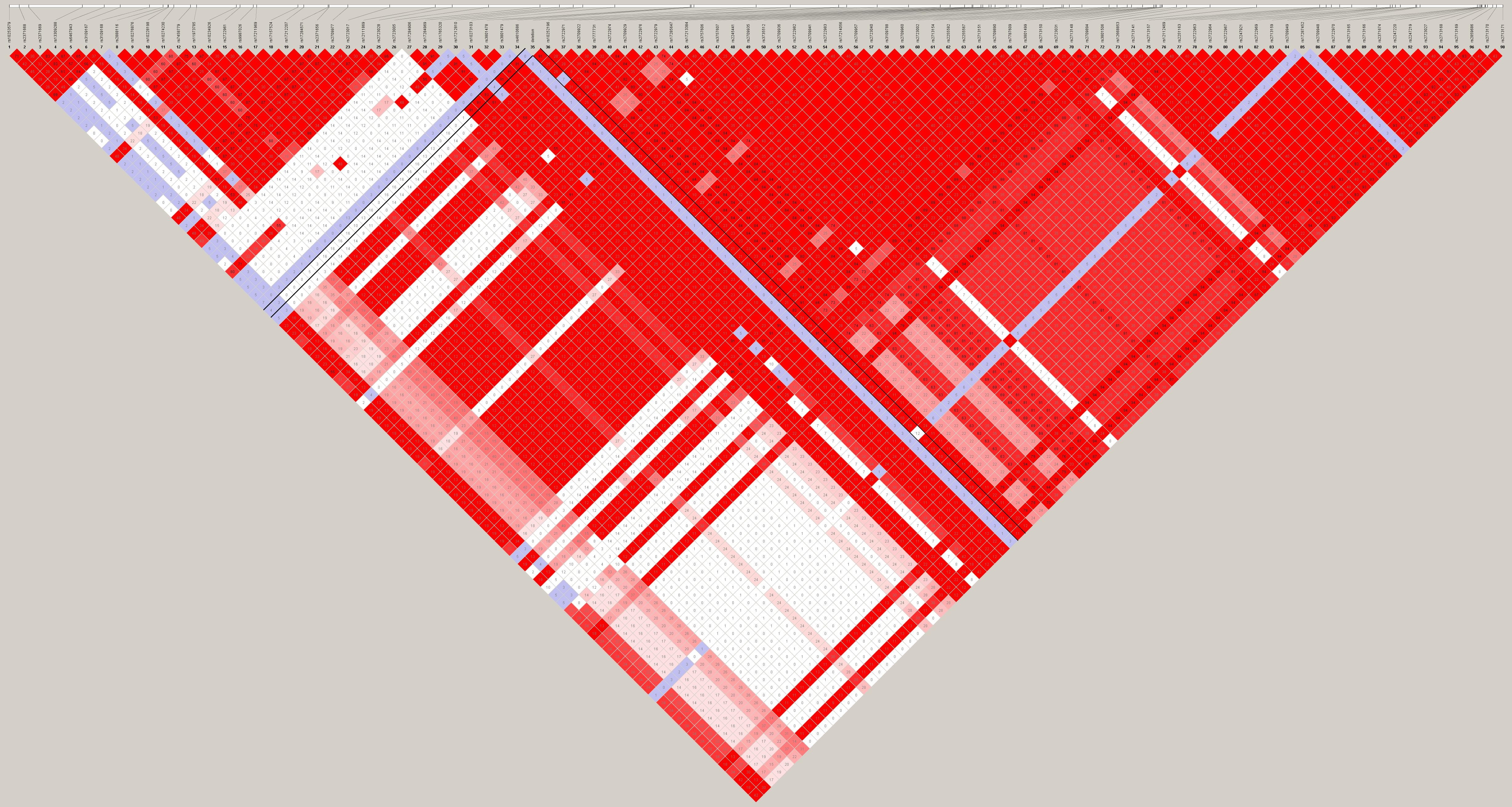

Supplement: Figure S5 — Plots show linkage disequilibrium (LD) of SNPs within 100 kb of each deletion. The default colouring scheme of Haploview is used, whereby positions are coloured white if LOD <2 and D' <1; blue if LOD <2 and D' = 1; shades of red as a function of D' if LOD≥2; bright red if D' = 1 and LOD ≥2. Numbers within the box refer to the r2 values between two given positions, and so are not directly connected to the colouring scheme. The solid black lines delineate the LD between the deletion and other markers in this region. a) Deletion at chr 1: 145,312,298–145,314,875; b) Deletion at Chr 2: 229,467,533–229,468,151; c) Deletion at Chr 3: 181,137,036–181,137,500; d) Deletion at Chr 4: 98,573,315–98,578,237; e) Deletion at Chr 5: 65,479,440–65,479,975; f) Deletion at Chr 5: 78,145,556–78,147,626; g) Deletion at Chr 6: 24,433,346–24,435,791; h) Deletion at Chr 6: 34,425,089–34,427,582; i) Deletion at Chr 6: 162,645,085–162,645,903; j) Deletion at Chr 7: 82,856,584–82,857,509; k) Deletion at Chr 12: 20,859,912–20,859,936; l) Deletion at Chr 14: 72,402,707–72,403,561; m) Deletion at Chr 14: 72,615,524–72,616,685; n) Deletion at Chr 15: 83,858,016–83,860,206; o) Deletion at Chr 16: 22,955,277–22,957,032; p) Deletion at Chr 16: 56,282,301–56,285,908; q) Deletion at Chr 16: 76,115,174–76,115,188; r) Deletion at Chr 16: 88,089,521–88,095,227; s) Deletion at Chr 19: 35,979,321–35,981,593; t) Deletion at Chr 22: 32,085,572–32,090,063. (8.90 MB ZIP) [file pone.0003104.s005.zip › SuppFig5/7_82856584_82857509_res.emphase.LD.PNG]

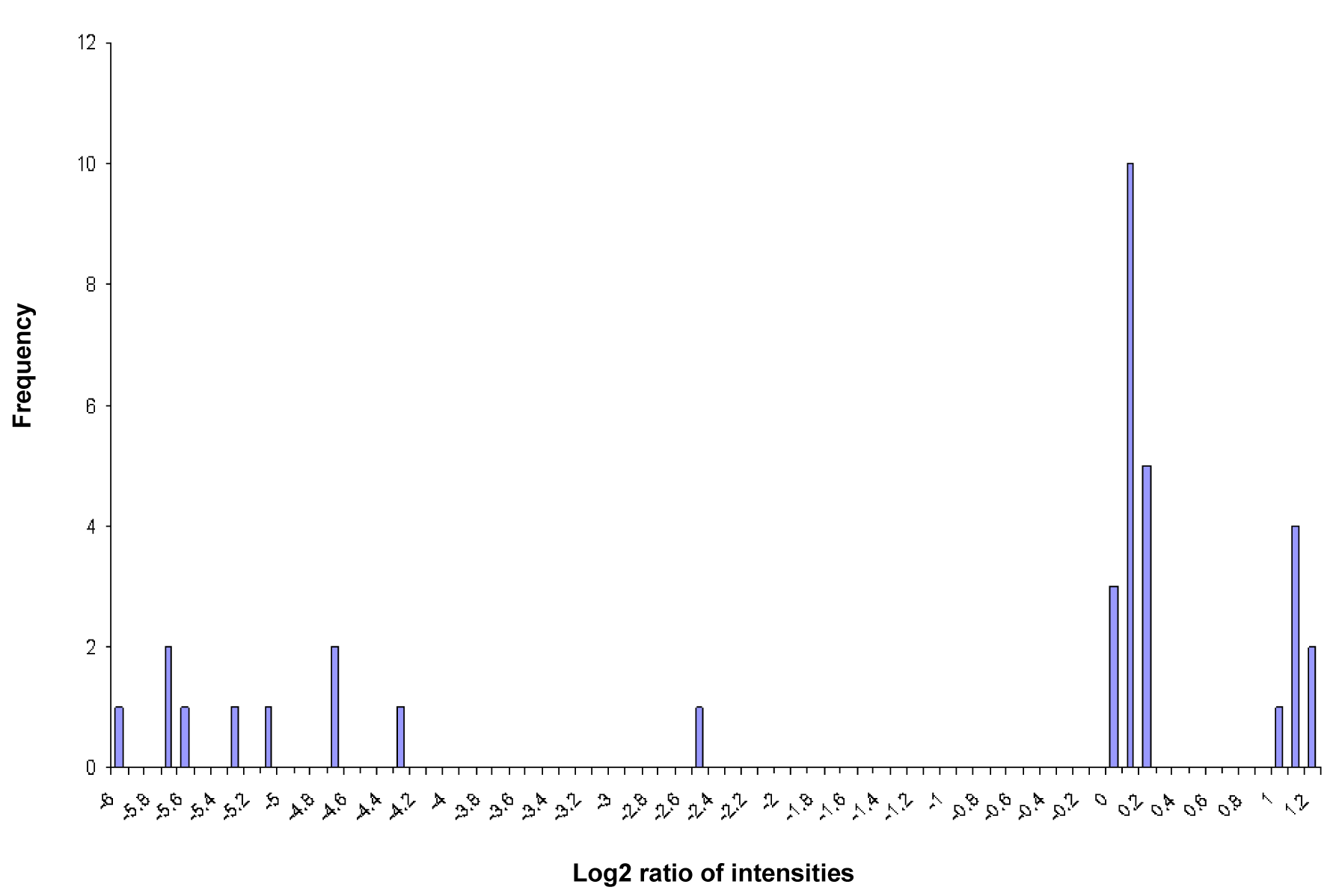

Supplement: Figure S6 — Histogram of Agilent aCGH log2 intensity ratios at probe located at chr 19: 35,979,761–35,979,820. With the assumption of a single copy deletion reference, the scores <2 are taken to be homozygous deletions; those with scores ∼0.0 are taken to be heterozygous deletions, and those with scores ∼1.0 are taken to be normal two copy samples. (0.09 MB TIF) [file pone.0003104.s006.tif]

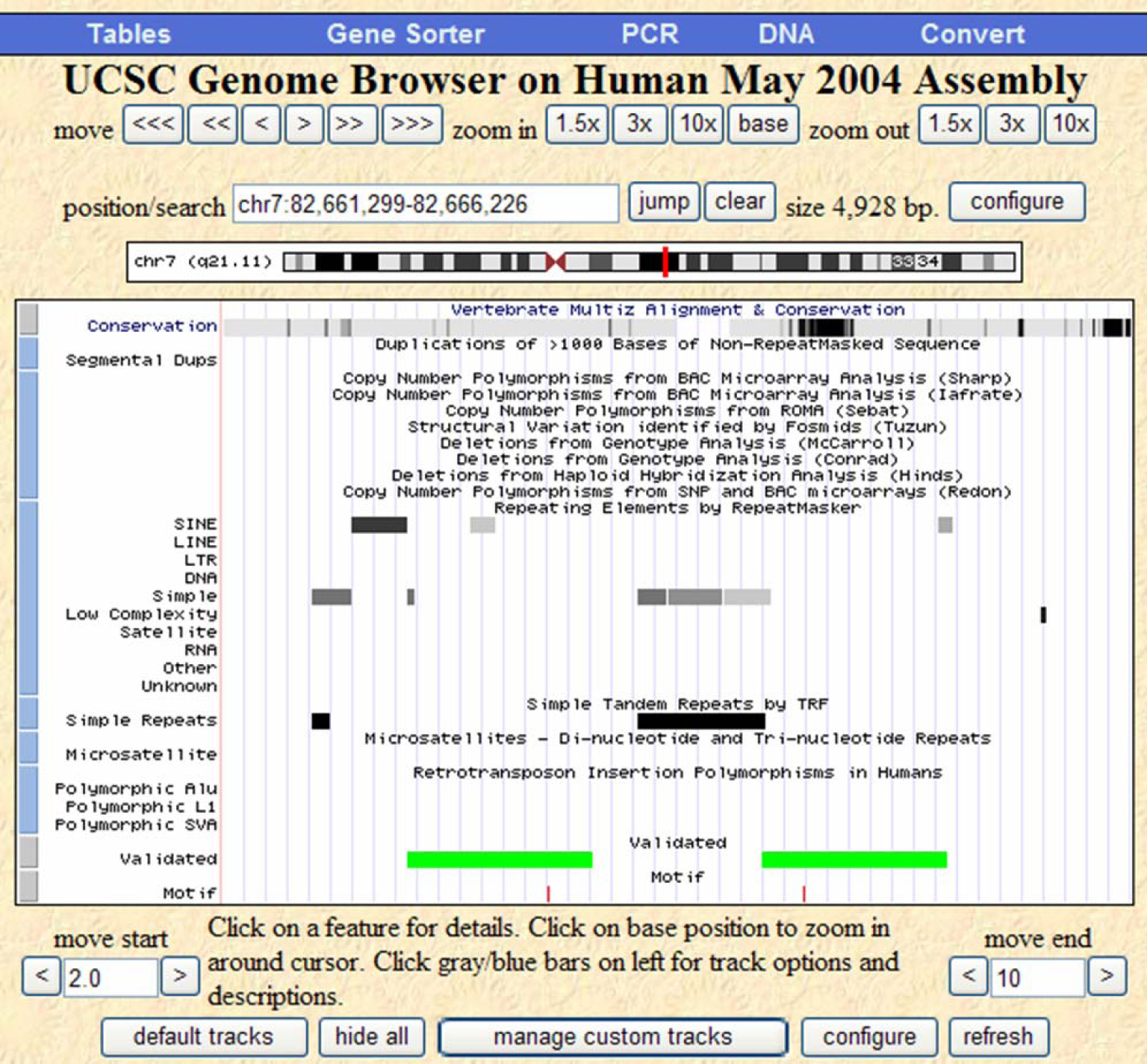

Supplement: Figure S7 — Example of UCSC Genome Browser representation of the genomic environment of the mapped breakpoints, showing motif sequence occurring in both flanking sequences of deletion. Using the custom track .bed files in Supplementary Tables 2 and 3, one can view 1 kb sequences flanking the breakpoints (green bars) together with the occurrences of the motif DHHACADGTG (red bars) and the repeat elements tracks (standard UCSC tracks). To generate the two custom tracks for this visual representation of the data, a user of the UCSC Genome browser would go to “manage custom tracks” (just under the genome view) and submit the .bed files. The repeat elements tracks need to also be turned on. (3.22 MB TIF) [file pone.0003104.s007.tif]

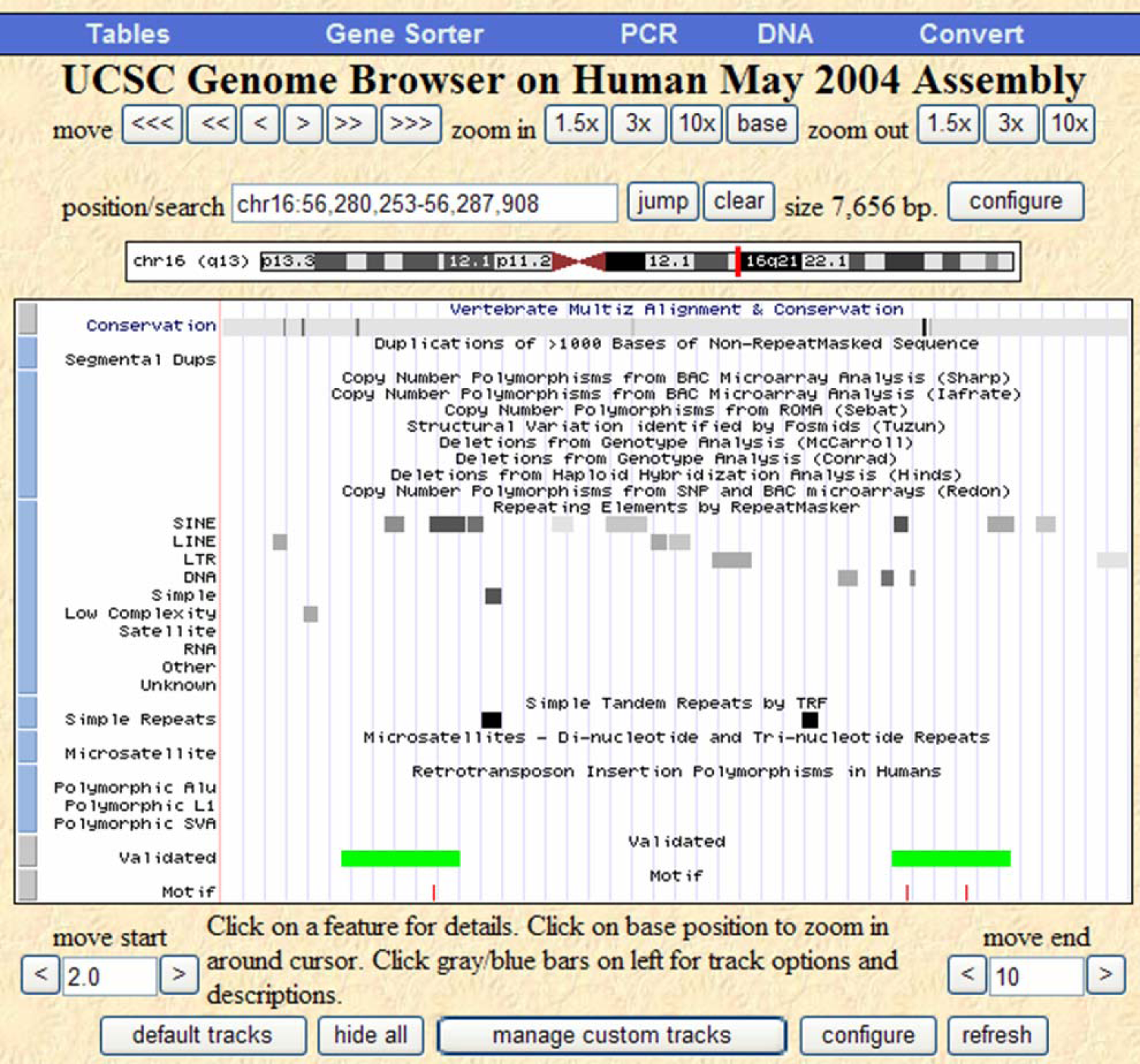

Supplement: Figure S8 — Same as Figure S7, but the motif can be seen to co-occur in repeat elements as well as in the flanking sequences on both sides. (3.24 MB TIF) [file pone.0003104.s008.tif]
