# Supplementary material for: Small Deletion Variants Have Stable Breakpoints Commonly Associated with Alu Elements
Source: PLoS One. 2008 Aug 29;3(8):e3104. doi: 10.1371/journal.pone.0003104 (PMC2518860; doi:10.1371/journal.pone.0003104)
Supplement: Figure S4 — DNA sequences at breakpoint junctions. Reference genome sequences spanning each breakpoint junction are shown aligned against each other according to the sequence present on chromosomes carrying deletions (highlighted in red). * indicates blocks of sequence identity/microhomology at the breakpoint junctions; + indicates further stretches of sequence similarity surrounding some breakpoint junctions. (0.03 MB DOC) [file pone.0003104.s004.doc]

**Figure S4**

chr1: 145312298-145314875

CAAAAAATATTTTTAAACTTAAAACTAATTTAAAAATTTA

GTTTGGTAATTTCATTGTCTTTTTTCTTTTTTTTTTTTTT

*

microhomology = 1

chr2: 229467533-229468151

GTGAGCCACCGCGCCCGGCCTCTCCCTTATTCTTCTTCCA

CATGTTGCCCCTCAGTGGCACACCCTCTGTATTTCATGTT

microhomology = 0

chr3: 181137036-181137500

TTTCTCCTTTATGTTGATTCTGAGTAACTTAATTCTGCCC

TCTTGTACTAAAACAGAATCAGTGGGGTATTAGAGTAGCA

**

microhomology = 2

chr4: 98573315-98578237

ATGAAAGACAACAAGTGTTGGTGAGGATAGAAAAAAGGGG

CAGGTGGAGCTGCCTGCCAGTCCCGTGCTGTGCGCCCACA

*

microhomology = 1

chr5: 65479440-65479975

TTTAAAAATAATTGTATAGTGCTATCATTATGTAATATAT

TTTTAAAAATATTGTATAGTGCTATCATTATGTTTAATAA

+++ ++++ ***********************

microhomology = 23

chr5: 78145556-78147626

CTATAAAACACAAGTACCAAACAGTCTCCAATAAAAACAC

GGTCTGAAGTCATTTCTTATGCCCTGGGTTATCATCTTCA

microhomology = 0

chr6: 24433346-24435791

AATCCAAAACCAGAATCTCCAGAGCCCCGTGGTCAAAACT

CCGGGTTCAAGCAATTCTCCTGCCTCAGCCTCCTGAGTAG

*****

microhomology = 5

chr6: 34425089-34427582

AGAAAGAAGAGTCTATAAGACCAAGTGACTGACAGGACGT

GAGTCCCCAGGGTGTACAGAGATTGCCTCTGTACAGTGCA

***

microhomology = 3

chr6: 162645085-162645903

ATTATATCTCAAATGGATATCATTACCTCTGTGAAGGG

TTTT

AACTCAAGTGAAAATGGAAATCCAGGTTTTTAGATGTAAT

microhomology = 0

chr7: 82856584-82857509

TGAATAATGGATAGGAATAAAATCAACAAAATGAGAAATG

CGCGTATATATGCGCGTATATATGCGCGTATATATGTGTA

*

microhomology = 1

chr12: 20859912-20859936

AGCATCAGCAACAATTAAAAATATTCACTTGGTATCTGTA

TAG

ATTCACTTGGTATCTGTAGTTTAATAATGGACCAACATCAACA

microhomology = 0

chr14: 72402707-72403561

CAGACTGAGGTCACCTCAGTCTGTTCCTGCCACAGCCTCC

GAGATGGGGTCTCACTGTGTTGTCCGGGTTGTTCTCAAAC

**

microhomology = 2

chr14: 72615524-72616685

CTTGTTGATTCACTTTTTTTACCTTTTTAAGATTTTATCT

TTACACATCTTTTTTTTTTTTTTTTTTTTTTTTTTTTGAG

******* +++++ ++++

microhomology = 7

chr15: 83858016-83860206

TTCTTTATCTTCTGTCTTTTCCGATTTTGAAGAATACAGT

AACAACTTCTTTAACATTTTAAACATCTTTTCTGTTGTCC

****

microhomology = 4

chr16: 22955277-22957032

GAGATTCTCAAATATGAAGGCTTATGCAAAAACATTA

GATTCT

CTGATTTACAGCAAGAGAAGAGGCAAATTGACACAAGAAGCTT

microhomology = 0

chr16: 56282301-56285908

><

TCTGTCTCTTAAAAAAAAAAAATATATCTAGGCATGGTAG

GTAAATAACCCCATGGCCAATTTTTTTTTTTTTTTTTTTT

**

microhomology = 2

chr16: 76115174-76115188

GGACTGTTGTGGGGTAGGGGGGAGCGGGGGGGGGGAGCGG

AGGGGGGAGCGGGGGGGGGGAGCGGGGAGGGATAGCATTG

+ ++++ **** + + ++ +++

microhomology = 4

chr16: 88089521-88095227

AATTTTTTTTTTTTTTTTTTTGAGACAGAGTCTAGCTCTG

ACAGAGCAAAATTCCGTCTCAAAAAAAAAAAAAAAAAATA

microhomology = 0

chr19: 35979321-35981593

CAAGATCGCACCAATGCACTCCAGCCTTGGTGACAATGTGAGACCCTTATCTTTTAAAAAAATAA

CAGGATTGCACCACTGCACTCCAGCCTGGGTGACAATGTGAGACCCTGTCTCAAAAGAAAAAAAA

++ +++ ++++++ ************* +++++++++++++++++++ + +++++ ++

microhomology = 13

chr22: 32085572-32090063

CATCTTTTTTTTTTTTTTTTTTTTTGAGATGGAGTCTCGC

CTTTGCTATATATTTTTTTTTTTTTGAGACGGAGTCTCGC

***************** ++++++++++

microhomology = 17
