# Supplementary material for: Small Deletion Variants Have Stable Breakpoints Commonly Associated with Alu Elements
Source: PLoS One. 2008 Aug 29;3(8):e3104. doi: 10.1371/journal.pone.0003104 (PMC2518860; doi:10.1371/journal.pone.0003104)
Supplement: Table S1 — Identification of extended deletion haplotypes and Hardy-Weinberg equilibrium. For each deletion, we identified the longest extended haplotype which was common to 100% of haplotypes with this deletion. We report the haplotype, the length of the haplotype. For the deletion on chromosome 19, we report the results of the calculation assuming a reference with 2 copies as well as the results assuming a reference with 1 copy (in brackets). (0.04 MB DOC) [file pone.0003104.s009.doc]

**Table S1– Identification of extended deletion haplotypes**

| **Genomic Position (from UCSC Genome Browser March 2006)** | **Sample Frequency (out of 50)** | **HWE p-value** | **Deletion haplotype (with HapMap imputed haplotypes)** | **Length of largest common haplotype (kb)** |
| --- | --- | --- | --- | --- |
| Chr 1: 145,312,298 - 145,314,826 | 0.06 | 1.0 | AAAABBBAABBBABABAAAAAABAABAABBABBABBBABBBBBABBBAABBAAABBBBBAAAAAAAAAABAABBBABBAABBBAAAABAABAABAABBABABAAABBABABBABABBABABBAAABBBABABABBBBAAABBBABBABBBBABBAAAABAABBBBAAAABBAABBAABAAABABAAABAABAAAABBBAAAAAABABAAAAAAAAABAAABAABABAABABBBBABABBBABAABBAAAABAAAABAABABABBABBBB_ABAABBBBABAABBBAAAAB | 234 |
| Chr 2: 229,467,528 - 229,468,147 | 0.20 | 1.0 | _BB | 1 |
| Chr 3: 181,137,036 - 181,137,512 | 0.18 | 0.05 | _ | - |
| Chr 4: 98,573,325 - 98,578,248 | 0.22 | 1.0 | BABBBBAAABBBBBBBBABAABBABAABBBABAABB_BABABBABB | 90 |
| Chr 5: 65,479,440 - 65,479,977 | 0.04 | 1.0 | - | - |
| Chr 5: 78,145,555 - 78,147,627 | 0.16 | 1.0 | AA_A | 7 |
| Chr 6: 24,433,341 - 24,435,786 | 0.18 | 0.88 | - | - |
| Chr 6: 34,425,054 - 34,427,548 | 0.08 | 1.0 | A_ABBAABBBB | 34 |
| Chr 6: 162,645,085 - 162,645,903 | 0.10 | 1.0 | BABBBABBABAAAABAABABBBABAABBBBBAABBABAABBAABBAAABBBBABBABBBBABBBBBAAB_ABBBAB | 73 |
| Chr 7: 82,856,584 - 82,857,511 | 0.24 | 0.62 | BAAABABBBA_AAAAABBBAAABBABABBABBBBBBBABABAAABABBAAABABAAABBBBAB | 57 |
| Chr 12: 20,859,912 - 20,859,937 | 0.52 | 0.70 | ABA_AAA | 12 |
| Chr 14: 72,402,705 - 72,403,559 | 0.10 | 1.0 | BBBAB_AABBBAABB | 15 |
| Chr 14: 72,615,517 - 72,616,679 | 0.20 | 1.0 | _AABA | 13 |
| Chr 15: 83,858,016 - 83,860,206 | 0.28 | 0.62 | _A | 1 |
| Chr 16: 22,955,277 - 22,957,032 | 0.88 | 1.0 | BBB_ABAAB | 4 |
| Chr 16: 56,282,253 - 56,285,908 | 0.36 | 0.85 | BBBBAABBABAABAAABAAAAABBAABB_AABBBBB | 33 |
| Chr 16: 76,115,174 - 76,115,189 | 0.10 | 1.0 | BABBABABBABA_ | 12 |
| Chr 16: 88,089,521 - 88,095,227 | 0.04 | 1.0 | N/A (only 1 sample with genotype data) | N/A |
| Chr 19: 35,979,346 - 35,981,619 | 0.54 | 2e-9 (1.0) | AB_BB | 8 |
| Chr 22: 32,085,565 - 32,090,046 | 0.16 | 1.0 | B_AAABAAAABAABBABBABBBAAABBBABBBBBABBBABBBBAABBA | 45 |
